# Supramolecular Chalcogen‐Bonded Shape Memory Actuators

**DOI:** 10.1002/anie.202508101

**Published:** 2025-06-01

**Authors:** Hongshuang Guo, J. Mikko Rautiainen, Hao Zeng, Kari Rissanen, Arri Priimagi, Rakesh Puttreddy

**Affiliations:** ^1^ Faculty of Engineering and Natural Sciences Tampere University P.O. Box 541 Tampere FI‐33101 Finland; ^2^ Department of Chemistry University of Jyvaskyla P.O. Box 35 Jyväskylä FI‐40014 Finland

**Keywords:** Actuator, Chalcogen, Chalcogen bond, Programmable, Shape memory, Stamping

## Abstract

Supramolecular liquid crystal elastomers (LCEs) incorporating chalcogen bonds (ChBs) have been synthesized and investigated for shape memory and actuation properties. LCEs featuring strong Se⋯N ChBs (−40 to −43 kJ mol^−1^) exhibit both shape memory effects (SME) and reversible actuation, while those with weaker S⋯N ChBs (−32 to −34 kJ mol^−1^) do not. The Se⋯N‐based LCEs transition from one‐way to two‐way SME after three days at room temperature, enabling programmable 2D/3D shape transformations and rewritable surface patterns. They also enable the fabrication of light‐powered crawling robots as well as temperature‐responsive knot‐like actuators. These results highlight the critical role of the ChB strength and specificity in designing functional materials. Solid‐state NMR, Raman spectroscopy, and density functional theory calculations provide further molecular‐level insights into ChB interactions, laying the groundwork for advanced supramolecular actuators and shape‐morphing LCEs.

## Graphical abstract

Supramolecular chalcogen‐bonded liquid crystal elastomers incorporating Se⋯N interactions exhibit distinct shape memory and actuation properties. Stronger Se⋯N ChBs enable programmable one‐way and two‐way SME, while weaker S⋯N ChBs fail to induce actuation. Solid‐state NMR, Raman spectroscopy, and DFT reveal the molecular interactions governing ChB‐driven mechanical responses.

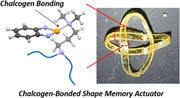

## Introduction

Non‐covalent interactions (NCIs) are fundamental to functions of both natural and synthetic materials, revolutionizing material design for self‐healing,^[^ ^1^ ^]^ dynamic,^[^ ^2^ ^]^ and programmable^[^ ^3^ ^]^ systems used in various applications, including molecular electronics^[^ ^4^ ^]^ and robotics.^[^ ^5^ ^]^ Even covalently bonded materials such as graphene,^[^ ^6^ ^]^ rely on one or more types of NCIs for structural integrity and functionality. Among the main NCIs are ionic interactions,^[^ ^7^ ^]^ van der Waals forces,^[^ ^8^ ^]^ and hydrogen bonding (HB),^[^ ^9^ ^]^ each contributing uniquely to self‐assembly, stability, and function. The HB, valued for its strength, specificity, and directionality, is central to intricate material designs. However, the effectiveness of HB‐driven materials is constrained by the “hard and soft acids and bases (HSAB)” principle,^[^ ^10^ ^]^ which dictates the affinities of HB donors and acceptors. HBs predominantly form between “hard” donors (e.g., N─H, O─H) and “hard” acceptors (e.g., O, N), leading to a natural preference for such interactions. This bias limits the incorporation of “soft” donor and acceptor species, which often interact weakly or are entirely disregarded in HB‐driven systems.^[^ ^11^ ^]^ Additionally, the complex interplay of multiple HBs and their (synergistic) interaction energies frequently outcompete donor–acceptor interactions of soft species,^[^ ^11^ ^]^ restricting the exploration of materials utilizing alternative NCIs beyond HB preferences.

**Figure 1:**
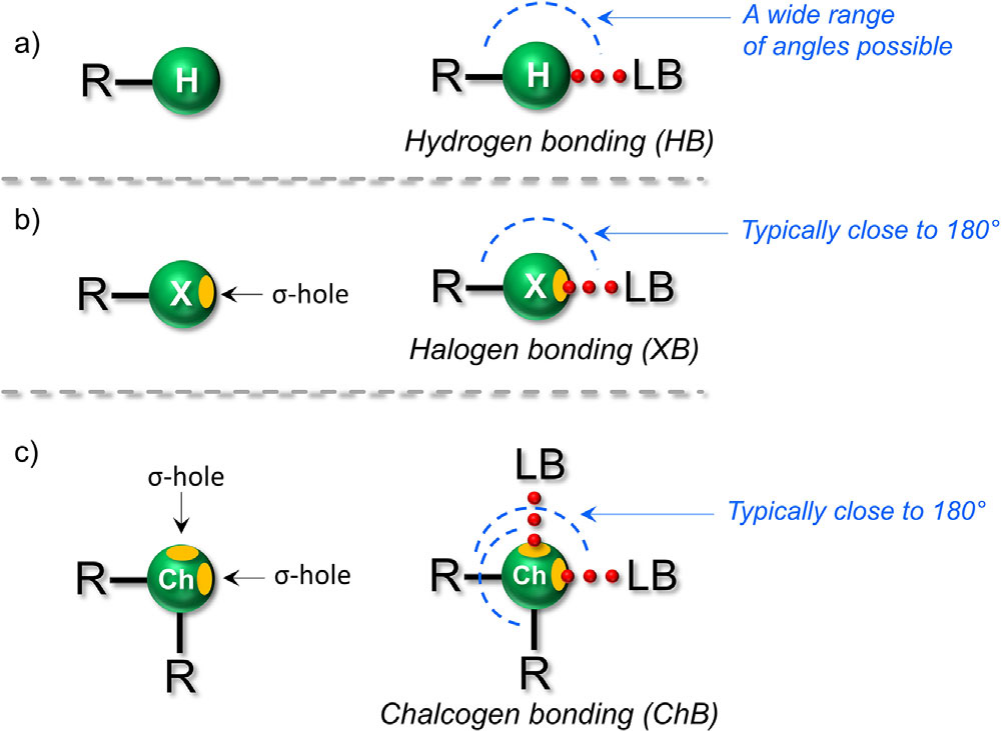
Comparison of non‐covalent interactions. General representation of a) hydrogen bonding (HB), b) halogen bonding (XB), and c) chalcogen bonding (ChB). Here, H = hydrogen, X = halogen, Ch = chalcogen, and LB = Lewis base. The orange‐colored ellipsoid shapes illustrate σ‐holes, and the red‐dotted lines represent non‐covalent interactions.

To address these limitations, σ‐hole interactions, such as halogen bonding (XB)^[^ ^12^ ^]^ and chalcogen bonding (ChB),^[^ ^13^ ^]^ have emerged as promising alternatives in material design. These NCIs occur between a Lewis acidic XB/ChB donor and a Lewis basic XB/ChB acceptor (Figure 1a–c). Unlike HBs, σ‐hole interactions originate from electron‐deficient regions along the extension of a covalent bond on halogen or chalcogen atoms (Figure 1a–c). While XB and ChB share geometric similarities, ChB is distinct in its ability to form bifurcated R─Ch⋯LB interactions (Ch = chalcogen, LB = Lewis base)^[^ ^14^ ^]^ due to the presence of two σ‐holes on chalcogens (Figure 1c). In contrast to HB, which is inherently limited to a single H‐atom, XB and ChB are versatile enough to involve various atoms,^[^ ^15^ ^]^ with I‐ and Br‐atoms serving as strong XB donors and Se‐ and Te‐atoms as strong ChB donors. The tunability of XB/ChB via different donor atoms allows for precise control over donor–acceptor pairing, optimizing material properties and functions. Both XBs and ChBs can deviate from linearity, but their directionality is generally more constrained and predictable than that of HBs, which are inherently more flexible due to the absence of filled p‐orbitals on the H‐atom and the involvement of the R─H σ* orbital.^[^ ^16^, ^17^, ^18^ ^]^ Over the past two decades, this predictable geometry has made XB a powerful tool to engineer molecules for applications in catalysis,^[^ ^19^ ^]^ biology,^[^ ^20^ ^]^ materials,^[^ ^21^, ^22^ ^]^ organic frameworks,^[^ ^23^, ^24^ ^]^ and polymers.^[^ ^25^ ^]^

Conversely, the potential of ChB remains underexplored, with studies specifically limited to catalysis,^[^ ^26^ ^]^ crystal engineering,^[^ ^27^ ^]^ and organic electronics.^[^ ^28^ ^]^ In the latter, achieving co‐planar aromatic assemblies is crucial for intermolecular charge‐carrier transport.^[^ ^29^ ^]^ This can be accomplished through two primary strategies: (i) covalently linking adjacent aromatic rings^[^ ^30^ ^]^ or (ii) “conformationally locking”^[^ ^31^ ^]^ via specific NCIs. The latter approach leverages heteroatoms such as oxygen and nitrogen alongside chalcogen atoms to induce S⋯O and S⋯N ChBs, effectively restricting C─C bond rotations and enhancing co‐planarity and solution processability.^[^ ^29^ ^]^ For example, Chen et al.^[^ ^32^ ^]^ introduced alkoxy and electron‐withdrawing ester substituents into polymers featuring thiophene donors and benzothiadiazole acceptors. The resulting S⋯O ChBs enforce backbone planarity, significantly impacting electronic properties. The ester‐substituted polymer exhibits a lower HOMO energy level (−5.2 eV) compared to its all‐alkoxy counterpart (−4.9 eV), increasing oxidation resistance. This stabilization was crucial for improving the open‐circuit voltage of the organic solar cell while maintaining a highly planar conformation through inter‐ring ChBs. Despite these advancements in (quasi) static polymers, integrating ChBs into dynamic, shape‐changing materials such as liquid crystal elastomers (LCEs)^[^ ^33^ ^]^ or polymer networks remains challenging. The proximity of two σ‐holes on chalcogens creates steric clashes that hinder the formation of optimal ChB donor–acceptor interaction.^[^ ^34^ ^]^ Furthermore, unlike XB, which forms directional single XB interactions, the dual σ‐holes on chalcogens can result in only one σ‐hole participating in ChB, or neither, resulting in unpredictable structural effects. These inconsistencies contribute to material fatigue, reduced stability, and compromised functional performance. Thus, can the ChB be exclusively attributed to macroscopic function?

**Figure 2:**
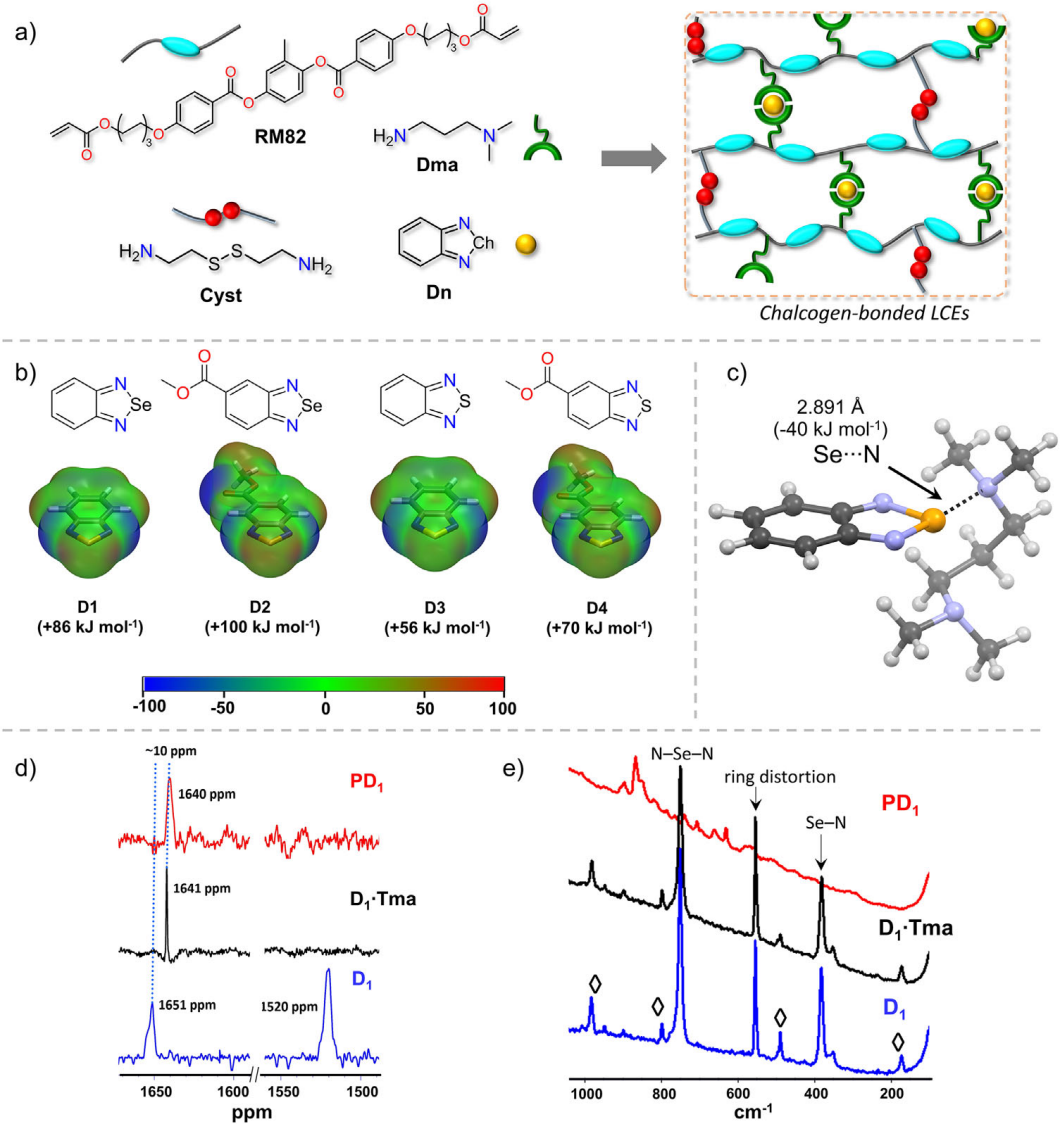
Synthesis of LCE, DFT calculations, and characterization. a) Chemical structures and the synthetic route to obtain chalcogen‐bonded LCEs, along with an illustration of the polymer network. b) Chemical structures of ChB donors and their DFT molecular electrostatic potential (MEP) surfaces; the color coordinates represent different bond energies. c) DFT‐optimized 1:1 **D_1_**:**Tma** chalcogen‐bonded complex, calculated at the M06‐2X/def2‐TZVP level of theory. d) SSNMR of **D_1_** (red trace), **D_1_·Tma** (black trace), and **PD_1_** (red trace). e) Raman spectra of **D_1_** (red trace), **D_1_·Tma** (black trace), and **PD_1_** (red trace).

In this study, we prepared chalcogen‐bonded LCEs by melting the mesogen **RM82**, cysteamine (**Cyst**), and *N*,*N*‐dimethylaminopropylamine (**Dma**) together with four different ChB donors containing either Se‐ or S‐atoms. The **RM82**, **Cyst**, and **Dma** undergo polymerization via the Aza–Michael addition reaction.^[^ ^35^ ^]^ Within these materials, the nitrogen in the ─N(CH_3_)_2_ group of tethered **Dma** acts as a ChB acceptor, forming Se/S⋯N interactions with Se‐ and S‐containing donors (Figure 2a). We investigated the importance and role of these ChBs in shape memory and actuation. Solid‐state nuclear magnetic resonance (SSNMR) and Raman spectroscopy are used to characterize the ChBs, density functional theory (DFT) to assess the donor–acceptor electronic structures and interaction energies, and mechanical properties testing to evaluate their performance.

## Results and Discussion

A total of six LCE materials, **P** (control), **PD_1_**, **PD_2_**, **PD_3_**, **PD_4_**, and **PChB**, were synthesized (Figures 2 and S1). **P** prepared using an **RM82**:**Cyst**:**Dma**:**D*_n_*** equivalent ratio of 1:0.3:0.4:0 without a ChB donor serves as the primary reference. **PD_1_**–**PD_4_** were synthesized using **D_1_**, **D_2_**, **D_3_**, and **D_4_**, respectively, using a **RM82**:**Cyst**:**Dma**:**D*_n_*** ratio of 1:0.3:0.4:0.2. Note that **D_1_** and **D_2_** contain Se‐atom as the chalcogen donor, whereas **D_3_** and **D_4_** contain S‐atom. The electron‐accepting ability of the Se‐atom in **D_1_**/**D_2_** and the S‐atom of **D_3_**/**D_4_** varies due to the presence of the electron‐withdrawing ester group. Additionally, **PChB** was synthesized as a secondary reference without **Cyst**, using **RM82**:**Cyst**:**Dma**:**D_1_** equivalent ratio of 1:0:1:0.5. Successful polymerization was confirmed by the FTIR, as evidenced by the disappearance of C═C stretching vibration peaks in the 1640–1680 cm^−1^ range, indicating complete monomer conversion into polymer chains, and the formation of ChB induces the shift of ─N(CH_3_)_2_ (1100–1300 cm^−1^, Figure S2), which is later also confirmed by SSNMR (Figure 2d).

DFT molecular electrostatic potential (MEP) surface analysis was conducted at the M06‐2X/def2‐TZVP^[^ ^36^, ^37^ ^]^ level of theory to evaluate the magnitude of σ‐hole (*V*_s,min_) on chalcogen atoms in **D_1_**–**D_4_** (Figure 2b and further details in Supporting Information). The σ‐hole magnitude is influenced by the size of the chalcogen, with the Se‐atom exhibiting a larger *V*_s,min_ than the S‐atom. Additionally, ester‐substituted ChB donors show larger *V*_s,min_ values than their unsubstituted benzene ChB donors. This increase is attributed to the electron‐withdrawing ester group, which lowers the LUMO energy level containing σ*‐orbital and enhances the chalcogen's electron acceptor ability.^[^ ^38^ ^]^ For instance, the *V*_s,min_ of the Se‐atom in **D_2_** is 14 kJ mol^−1^ larger than in **D_1_**, indicating that **D_2_** can form stronger Se⋯N interactions compared to **D_1_**. In the polymerization process, a Michael addition occurs between the acrylate group of **RM82** and the ─NH_2_ group of **Dma**, resulting in the formation of a *tert*‐amine group within the LCE chain. This *tert‐*amine group and the ─N(CH_3_)_2_ group are separated by a ─(CH_2_)_3_─ spacer. To estimate the interaction energies (Δ*E*_int_) of Ch⋯N ChBs, *N*,*N*,*N*,*N*‐tetramethylpropane‐1,3‐diamine (**Tma**), which possesses two *tert*‐amine groups separated by a ─(CH_2_)_3_─ spacer, was employed as a ChB acceptor. The Δ*E*_int_ values for the 1:1 **D*_n_***:**Tma** adducts were computed at the M06‐2X/def2‐TZVP level of theory (Table 1). The results indicate that Se⋯N ChBs exhibit larger interaction energies than their S⋯N counterparts, consistent with the MEP values and comparable to literature reports on Se⋯N ChBs energies.^[^ ^30^ ^]^ This correlation reinforces the understanding that stronger σ‐holes lead to more robust ChB interactions.

**Table 1:** DFT‐optimized structural data.

Code	Ch⋯N (Å)	Δ*E*_int_ (kJ mol^−1^)
**D_1·_Tma**	2.891	−40.2
**D_2·_Tma**	2.874	−42.4
**D_3·_Tma**	2.933	−31.7
**D_4·_Tma**	2.913	−34.3

Computed chalcogen bond energies of **D*_n_*** ·**Tma** complexes at M06‐2X/def2‐TZVP level of theory (Ch = S, Se).

The glass transition temperatures (*T*_g_) of polymers were measured using differential scanning calorimetry (DSC). The *T*_g_ values of **P**‐**PD_4_** range from −3.1 to 4.3 °C and are consistently smaller for ChB donor‐containing LCEs (see Table 2 and Figure S3). Despite differences in the chemical composition of ChB donors, the *T*_g_ values among these LCEs do not differ significantly. This suggests that ChB donors increase the free volume between polymer chains, allowing greater chain mobility and thereby lowering *T*_g_ compared to reference **P**. The presence of Se‐based **D_1_** and **D_2_** donors, which form Se⋯N crosslinks, results in slightly reduced chain mobility compared to S‐based **D_3_** and **D_4_** donors with S⋯N crosslinks. This trend is reflected in the larger *T*_g_ values of **PD_1_** and **PD_2_** relative to **PD_3_** and **PD_4_**, underscoring the role of stronger Se⋯N interactions in modulating chain mobility. **PChB** synthesized without **Cyst** exhibited a dramatic *T*_g_ reduction from −1.0 to −18 °C (Figure S3), demonstrating the pronounced effect of ─S─S─ bonds or S‐atoms capability to form a wide range of NCIs such S⋯H and S⋯O═C^[^ ^39^ ^]^ on glass transition temperature. Note that *T*_g_ values are highly sensitive to chemical structure and the type of NCIs present. Strong HB‐driven materials, for example, typically exhibit larger *T*_g_ values. Cortes et al.^[^ ^40^ ^]^ reported *T*_g_ values for a series of polyketone polymers. In their study, a polyketone polymer with a hexyl chain had a *T*_g_ of ∼ −15 °C, while a polymer with a hexane‐carboxylic acid group exhibited a *T*_g_ of +15 °C. The lower *T*_g_ of the hexyl‐based polymer was attributed to the flexible aliphatic side chain, which allowed greater segmental motion. In contrast, the hexane‐carboxylic acid group introduced strong HB, increasing the energy required for molecular relaxation and restricting polymer chain mobility.

**Table 2:** Chalcogen‐bonded material properties.

Code	*E*_Y_ (MPa)	*σ*_max_ (MPa)	*Ɛ*_max_ (%)	*T*_g_ (°C)
**P** (control)	2.0	1.7	270	4.3
**PD_1_**	2.5	4.0	210	−0.8
**PD_2_**	2.1	2.3	230	0.5
**PD_3_**	1.7	2.3	280	−3.1
**PD_4_**	1.7	2.9	230	−2.3

The glass transition and mechanical properties of **P** and **PD_1_**–**PD_4_**.

The mechanical properties of **P**‐**PD_4_** were evaluated by uniaxial stretching of LCE strips (dimension: 16 × 2 × 0.5 mm^3^) (Figure S4). The reference **P** demonstrates an elastic modulus (*E*_Y_) of 2.0 MPa and a tensile strength (*σ*_max_) of 1.7 MPa. The **PD_1_**‐**PD_4_** showed similar mechanical performance, with **PD_1_** demonstrating a larger tensile strength. These values are comparable to those reported for HB‐based LCEs.^[^ ^41^ ^]^ Mechanical testing is crucial for identifying strain and stress at break, which helps determine the optimum safe strain applicable during deformation in shape memory tests. Among the tested materials, the reference **P** and the S‐containing **PD_3_** and **PD_4_** samples emerge as the extensible networks, achieving elongation at break (*Ɛ*_max_) of 270%, 280%, and 230%, respectively. These materials are soft and highly deformable. Conversely, the Se‐based **PD_1_** (210%) and **PD_2_** (230%) exhibit smaller *Ɛ*_max_ compared to **P** and **PD_3_** but are still close to **PD_4_**. Despite the reduced elongation, they can undergo substantial deformation without tensile failure (Table 2). These textural differences are attributed to stronger Se⋯N crosslinks in **PD_1_/PD_2_**, compared to weaker NCIs (e.g., H⋯O, π–π, or S⋯N) in **P**, **PD_3_**, and **PD_4_**. While the soft **P**, **PD_3_**, and **PD_4_** films are unsuitable for shape memory studies, **PD_1_** and **PD_2_** exhibit effective shape memory properties. Cyclic tensile tests further support the material performance differences. For instance, in their unaligned state (Figure S5a,b,d,e), both **PD_1_** and **PD_3_** display typical hysteresis loops, indicating energy dissipation. Upon alignment (Figure S5c,f), **PD_1_** exhibits increased stiffness and reduced hysteresis, suggesting improved molecular orientation driven by stronger Se⋯N interactions. **PD_3_**, due to its weaker S⋯N interactions, does not show any data after alignment. The first and second cycles (Figure S5a–c) further highlight mechanical relaxation, especially in aligned **PD_1._** Nevertheless, based on the mechanical performance, **PD_1_** exhibits superior *E*_Y_ and *σ*_max_ properties and is therefore selected for further testing, including shape memory and actuator demonstrations.

Solid‐state ^77^Se NMR was employed to investigate Se⋯N ChBs in **PD_1_**. The ^77^Se SSNMR of **D_1_** reveals two distinct chemical shifts at 1651 and 1520 ppm (Figure 2d), indicating the presence of two non‐equivalent **D_1_** donors within the bulk sample, stabilized by Se⋯N ChBs among **D_1_** molecules. This observation is consistent with the ^77^Se SSNMR profile of the well‐documented 3,4‐dicyano‐1,2,5‐selenodiazole ChB donor, which exhibits chemical shifts at 1592 and 1548 ppm, corroborated by an X‐ray crystal structure showing two crystallographically independent molecules in its asymmetric unit.^[^ ^42^, ^43^ ^]^ The ^77^Se SSNMR spectra for **D_1_** in **D_1_** ·**Tma** and **PD_1_** complexes present a single signal at 1641 and 1640 ppm, respectively. These upfield chemical shifts, differing by ∼10 ppm from those of pure **D_1_**, suggest that only one type of **D_1_** exists in their bulk samples. Additionally, the upfield chemical shifts indicate the formation of Se⋯N ChBs between the Se‐atom of **D_1_** and the nitrogen of ─N(CH_3_)_2_ group in **Dma**/LCE‐**Dma**.^[^ ^44^ ^]^

Raman spectroscopy was used to further characterize **P**, **D_1_** ·**Tma**, and **PD_1_** (Figure 2e), focusing on Se─N covalent bond vibrations in the five‐membered selenodiazole ring to monitor the Se⋯N chalcogen bonding. In the spectrum of **D_1_** (blue trace), signals at 752 and 385 cm^−1^ correspond to the bending (or scissoring) of N─Se─N covalent bonds and symmetric stretching of Se─N covalent bonds, respectively.^[^ ^45^, ^46^ ^]^ Additional weak bands, marked with “◊” in Figure 2e, arise from “in‐plane” and “out‐of‐plane” vibrations involving C─C/N bonds in the ring (for more details, see Table S2).^[^ ^45^, ^46^ ^]^ Upon complexation with **Tma** (black trace), these signals remain unchanged, indicating minimal structural and electronic perturbation. This stability likely results from strong packing forces in the polymeric structure and sustained π‐delocalization between the five‐ and six‐membered rings. In contrast, these signals are absent in **PD_1_**, suggesting a loss of the characteristic selenodiazole vibrations. This behavior contrasts with the C─I bond stretching observed in (perfluoroiodobenzenes)C─I⋯N(pyridine) halogen‐bonded complexes, which typically exhibit redshift changes of 7–15 cm^−1^.^[^ ^47^ ^]^ In the DFT Raman spectra of **D_1_** and **D_1_** ·**Tma**, the C═N stretching modes overlap with the phenyl ring modes, making them indistinct (Figures S15–S18). However, the bending and stretching modes of the N─Se─N and Se─N covalent bonds in DFT‐computed **D_1_** and **D_1_** ·**Tma** complex appear at 791 and 391 cm^−1^, respectively.

**Figure 3:**
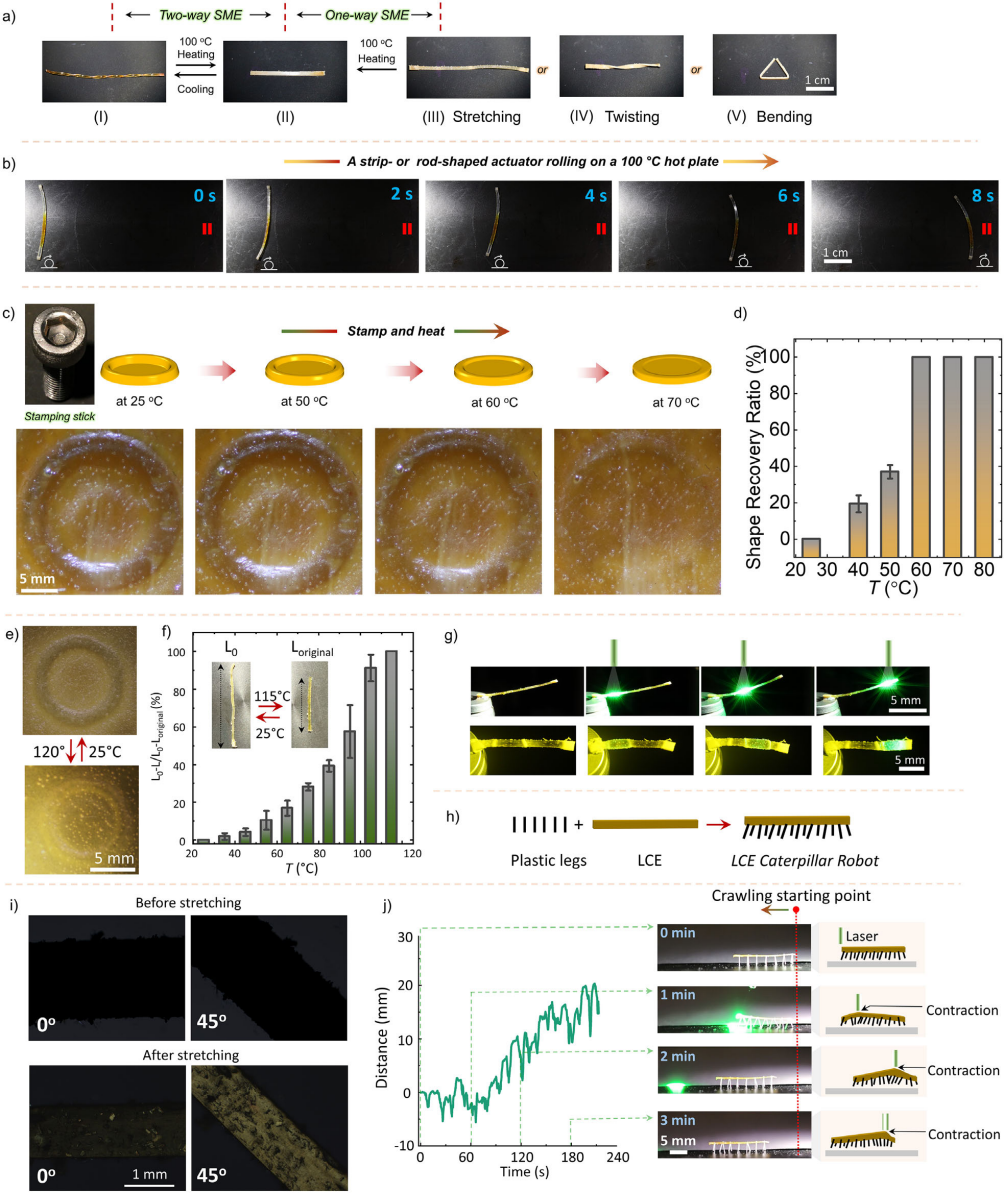
Shape memory, actuation, and light‐fueled robotic studies. a) One‐way and two‐way shape memory demonstration of **PD_1_**. b) Photographs of an actuator captured during the strip's rolling process on a 100 °C hot plate. The rolling direction is towards the red double pillars. c) Schematics and photographs illustrate the stamp‐and‐heat one‐way shape memory property. d) The chart presents the shape memory recovery ratio at different temperatures. e) Photographs of the reversible shape‐changing property of **PD_1_**’s stamp. f) The contraction under uniform heating. Inset photographs show the contraction upon heating and the reversible process upon cooling. L_0_ stands for the initial length (after stretching) and L is the contracted length under heating. g) Photographs of photothermal actuation of the **PD_1_** (534 nm, laser 250 mW). h) The schematic diagram details the preparation of the crawling robot. i) Polarized optical micrographs of the stretched **PD_1_** at 0° and 45° angles between the molecular director and the polarizer/analyzer before and after programming. j) The crawling robot's motion mechanism is elucidated, depicting its light‐fueled walking distance along the front side of the robotic structure under modulated light stimulation, accompanied by photographs at each position (laser 534 nm, 250 mW). All error bars reflect the outcomes of three independent experiments.

A temporary shape is programmed by stretching a **PD_1_** strip (dimension: 20 × 2 × 0.5 mm^3^) at ≥70 °C and subsequently cooling it to room temperature under the external force (Figures 3a I and S6). Upon reheating to 100 °C, the strip returns to its original shape (Figure 3a II). Other deformed configurations, such as twisted sticks and bent triangles (Figures 3a III–V), similarly recover to their original shapes upon heating at 100 °C. This one‐way shape memory effect is driven by the dynamic ChB network. At high temperatures, the ChB interactions become reversible, allowing reshaping. Upon cooling, the ChB network reforms, locking the temporary shape by storing strain energy. Reheating disrupts these bonds enabling the material to return to its original shape, maintained by disulfide crosslinks that act as a stable structural framework throughout the cycle.

The **PD_1_** exhibits reversible two‐way shape memory behavior through heating–cooling cycles without external force, provided the strip remains at room temperature for 3 days (Figures 3a I–II).^[^ ^22^ ^]^ This reversibility enables applications such as a rolling thermo‐actuator operating over a 100 °C hot plate (Figure 3b and Video S1). The shape memory effect arises from two functional domains within **PD_1_**: the LCE‐based actuation domain, responsible for crystallization‐induced elongation (CIE)^[^ ^48^ ^]^ and a structural domain with dynamic Se⋯N ChBs. Upon heating, the ChBs generate internal stress that activates CIE even under external stress‐free conditions. This allows cyclic melting and crystallization of the actuator domain, driving the reversible shape change.

The delayed onset of reversible actuation in **PD_1_** is primarily due to the relaxation of internal stresses and structural inhomogeneities, a behavior typical of certain LCE systems.^[^ ^49^ ^]^ Stretching at elevated temperatures disrupts the Se⋯N ChBs, leading to internal stress accumulation upon cooling. These stresses gradually relax over several days, as observed in the slow contraction of the **PD_1_** strip (Figure S7a). FTIR spectroscopy confirms the ChB disruption and reformation, that is, immediately after stretching and cooling, the *N*,*N*‐dimethylamino stretching frequency appears at 1248 cm^−1^, indicating ChB disruption. Over time, this peak shifts towards a higher frequency, suggesting the reformation and strengthening of ChBs (Figure S7b,c). In contrast, materials without strong ChB interactions (e.g., **PD_3–4_**) show no reversible actuation, even after prolonged equilibration, underscoring the critical role of ChBs. Mechanical testing further supports this conclusion. The **PD_1_**’s fracture strain increases from 52% to over 70% after 3 days, consistent with ChBs recovery (Figure S7d). In contrast, **PD_3_** lacking stronger ChBs’ interactions shows a gradual decline in Young's modulus over time (Figure S7e). These findings highlight the importance of dynamic ChBs in enabling structural reorganization and reversible actuation.

**PD_1_** also demonstrates 2D surface shape memory functionality (Figure 3c). By stamping a thin film (dimension: 20 × 20 × 0.5 mm^3^) at ≥70 °C and cooling it, an imprinted temporary shape is preserved. When reheated, the film recovers fully at >70 °C (Figure 3d). This process is erasable and rewritable (Figure S8). Similar to the two‐way SME of Figure 3a, the imprinted 2D surface retains its topographical structure at 27 °C for 3 days, allowing reversible two‐way SME between 27 and 120 °C with actuation occurring at 120 °C (Figure 3e). Such reversibility has the potential for stress‐free SMP products inspired by, e.g., Amazonica leaves, which change shapes with varying water temperatures. Additionally, diverse surface structures can be programmed (Figure S9). Thermal actuators use thermally induced elastic deformation and mechanical contraction as driving forces. **PD_1_**’s contraction rate was analyzed concerning temperature, showing contraction opposite to elongation (Figure 3f). The actuation speed increases with temperature, reaching 100% contraction at >110 °C. This suggests internally generated strain facilitated by a palette of NCIs such as (alkyl/aryl)C─H⋯O(carbonyl/ester/ether), π–π, and dynamic Se⋯N ChBs, which enhance the molecular packing. Materials with directional NCIs, like **PD_1_**, exhibit high contraction rates due to their specific molecular interactions. This observation highlights the essential role of Se⋯N ChBs and explains why weaker and less directional S⋯N ChBs‐based polymers do not exhibit comparable features.

Disulfide (─S─S─) bonds^[^ ^50^ ^]^ serve not only as dynamic covalent linkages capable of reversible bond exchange under exposure to heat, UV light, and redox reactions, imparting self‐healing and recyclability, but also exhibit photothermal conversion due to their light absorption characteristics. This dual functionality contributes to the light‐responsive behavior of **PD_1_**. Leveraging this, we developed a light‐controlled soft robot driven by the photothermal effect of ─S─S─ bonds.^[^ ^51^ ^]^ The **PD_1_** strip shrinks upon light exposure, reinstating its ordered (uniaxial) molecular alignment in the stretching direction (Figures 3g and S10). Polarized optical micrographs (POM) taken at 0° and 45° using a polarizer and the molecular director confirm this alignment compared to the pristine **PD_1_** in Figure 3i. By attaching plastic legs to the **PD_1_** LCE strip, we created a light‐powered caterpillar robot (Figure 3h). Upon exposure to laser light, the strip contracts at the irradiated area, enabling crawling motion (Figure 3j and Video S2). The caterpillar traverses ∼20 mm in 3 min, comparable to previously reported light‐fueled crawling robots (∼0.3−1 cm min^−1^)^[^ ^52^, ^53^, ^54^ ^]^ based on liquid crystal systems, demonstrating its effectiveness in light‐responsive actuation applications. The reference **P**, containing ─S─S─ bonds but lacking Se⋯N crosslinks, does not exhibit similar crawling motion, highlighting **PD_1_** ‘s unique mechanism relying on synergistic NCIs and dynamic Se⋯N crosslinks.

**Figure 4:**
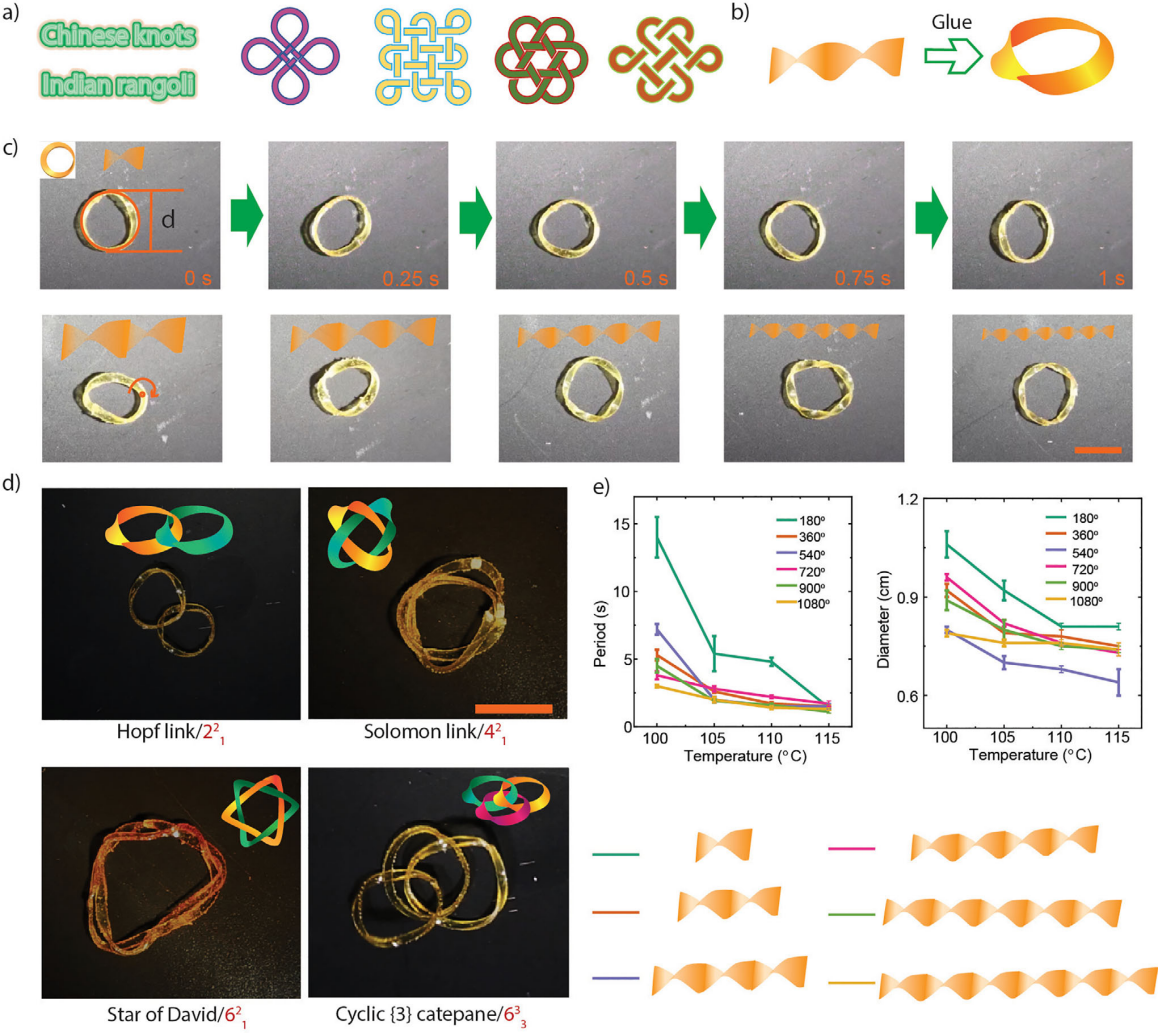
Chalcogen‐bonded knot actuation studies. a) Traditional patterns inspired by Chinese knots and Indian Rangoli, illustrating weaving techniques commonly used in everyday life. b) A schematic illustration of the preparation process for a single knot loop. c) Photographs showing the self‐rotating process of a single‐twist knot on a 100 °C hot plate, along with the knot structures featuring different numbers of twists. The inset defines the diameter, the number of twisting cycles, and the rotation period. d) Photographs showing different methods for connecting loop structures. e) Changes in the rotation period and loop diameter of a single knot structure under varying temperatures, and the schematic illustration of the twist method. All scale bars represent 1 cm, and all error bars indicate results from three independent experiments.

**PD_1_** was explored as an actuator for ring and knot structures through a programming approach (Figure 4a).^[^ ^55^, ^56^, ^57^, ^58^, ^59^ ^]^ These ring and knot structures were made by twisting pre‐programmed **PD_1_** strips and joining their ends using adhesive (Figure 4b). A full twist is defined as one 180° rotation before sealing the ends, and the number of twists was controlled by adjusting the degree of rotation. When the knot rings are placed on a 100 °C hot plate, these knot rings exhibit automatic rotation (Figures 4c,d, S11 and S12; Videos S3 and S4), with increasing speed as the temperature increases. Additionally, the structures contract, reducing the diameter and further enhancing their motion (Figure 4e). As the temperature increases, the strip forming the ring contracts, leading to a smaller decrease in diameter (Figure 4e). The period of rotation, i.e., the time required for a point on the strip to complete one full (360°) rotation, also decreases with rising the temperature. This is due to the material's rapid response to heat, which causes faster contraction and flipping, ultimately accelerating rotation. As shown in Figure S12b, the relationship between complex knot structures and the rotation period follows a trend where the period first increases and then decreases with the number of knots. More complex knot structures, including the Hopf link, Solomon link, Star of David, and catenane knots, also show automatic rotation on the hot plate (Figures 4d, S13 and S14; Video S5). The rotation period of the link and its relationship with the number of links is shown in Figure S14. As the number of link connections increases, the rotation period shortens. This is because the contact points between the two rings increase, enhancing overall friction, which in turn increases the rotation speed and shortens the period. Similar to single‐knot ring structures, their rotation speed increases with temperature while their diameter progressively decreases. Here the link number is defined as the number of contact points between the two rings. At the same temperature, a higher twisting angle results in a shorter rotation period (Figure 4e). This is because a greater twisting degree increases the number of contact points between the knot ring and the heated plate, the number of contact points, defined as the number of locations of the strip touching the surface, was influenced by the number of twists. While twists exceeding 360° followed a predictable pattern, even a 180° twist resulted in two contact points. As a result, a better definition of contact point refers to the locations where the twisted sections physically interact with the surface.^[^ ^60^ ^]^ A higher number of contact points enhanced both friction and heat transfer, leading to increased rotation speed (Figure 4e). These findings demonstrate the versatility of chalcogen‐bonded materials for programmable actuation highlighting the potential for intricate topological actuator applications.

The incorporation of ChB into LCEs marks a significant breakthrough in dynamic polymer design. While HB LCEs exhibiting 30%–50% actuation strain through hydroxyl,^[^ ^61^ ^]^ thiourethane,^[^ ^62^ ^]^ carboxyl,^[^ ^63^ ^]^ or UPy^[^ ^41^, ^64^ ^]^ interactions require high programming temperatures (generally above 100 °C) due to their strong bonding, and XB^[^ ^22^ ^]^ systems achieve higher strain (60%) at lower temperatures (60 °C) through weaker interactions, our ChB‐based LCE demonstrates unique advantages. With comparable actuation strain (∼50%), the intermediate bond strength of ChB (between XB and HB) enables the first successful integration of reversible actuation with unidirectional shape‐memory effects in LCEs. This novel dual functionality stems from the synergistic combination of a disulfide covalent network (providing structural memory) and a dynamic ChB network (enabling reversible deformability), thereby significantly expanding LCE application potential and establishing ChB as a powerful tool for developing tunable, reprogrammable responsive materials.

## Conclusion

In conclusion, we demonstrate chalcogen bonding as a simple yet effective strategy for designing shape‐changing and actuating LCEs with programmable properties. Although the LCEs composed of RM82, cysteamine, and *N*,*N*‐dimethylaminopropylamine contain numerous weak C─H⋯H─C hydrogen bonds (∼4 kJ mol^−1^ each) and covalent crosslinks, cumulatively exceeding the energy of Se⋯N chalcogen bonds, it is the Se⋯N interaction that uniquely drives the material functionality. The system remains unresponsive to weaker S⋯N interactions, highlighting how chalcogen bonding from a single molecule can specifically influence macroscopic functionality. This finding is significant given that the LCEs inherently exhibit complex and unknown supramolecular structures at the nanoscale, which typically obscure the direct structure‐function relationships. Additionally, the system is designed for adaptability, enabling researchers to expand its shape memory and actuator functions simply by substituting one ChB donor for another. Notably, this modularity facilitates the easy integration of new functionalities without the need for complete system redesigns or the use of intricate chalcogen bond donors, ensuring that the platform remains relevant as technology advances. This material has potential for advanced functional applications, including vapor‐responsive robots, artificial muscles, and sensors, by incorporating stronger Se⋯N or Te⋯N chalcogen bonds, or a combination of both.

## Author Contributions

H.G. and R.P. are responsible for project conception, design, experiments, and manuscript preparation. J.M.R. carried out the computational studies. H.Z. proposed the stamp conception. A.P. and K.R. provided the infrastructure resources for the project. All authors read and approved the manuscript.

## Conflict of Interests

The authors declare no conflict of interest.

## Supporting information



Supporting Information

Supporting Information

Supporting Information

Supporting Information

Supporting Information

Supporting Information

## Data Availability

The data that support the findings of this study are available from the corresponding author upon reasonable request.
