# Supplementary material for: Supramolecular Chalcogen‐Bonded Shape Memory Actuators
Source: Angew Chem Int Ed Engl. 2025 Jun 1;64(30):e202508101. doi: 10.1002/anie.202508101 (PMC12281081; doi:10.1002/anie.202508101)
Supplement: Supplementary file 6 — Supporting Information [file ANIE-64-e202508101-s001.docx]

Supporting information for

**Supramolecular Chalcogen-Bonded Shape Memory Actuators**

Hongshuang Guo,*^[a]^ J. Mikko Rautiainen,^[b]^ Hao Zeng,^[a]^ Kari Rissanen,^[b]^ Arri Priimägi,^[a]^ Rakesh Puttreddy*^[b]^

^[a]^ Faculty of Engineering and Natural Sciences, Tampere University, P.O. Box 541, FI-33101 Tampere, Finland.

^[b]^ Department of Chemistry, University of Jyvaskyla, P.O*.* BOX 35, FI-40014, Jyväskylä, Finland.

Correspondence to: [hongshuang.guo@tuni.fi](mailto:hongshuang.guo@tuni.fi), rakesh.r.puttreddy@jyu.fi.

This PDF file includes:

Materials and Methods

Figures S1 to S14

DFT details and the xyz coordinates

References

Captions for Movies S1-S5

**Additional supplementary material for this manuscript includes:**

Movies S1-S5

**Materials and Methods**

1,4-Bis-[4-(6-acryloyloxyhexyloxy)benzoyloxy]-2-methylbenzene (99%, RM82) was purchased from SYNTHON Chemicals GmbH & Co. 1-Amino-3-(dimethylamino)propane and cystamine dihydrochloride were obtained from TCI Chemical Europe. Cystamine was prepared by treating cystamine dihydrochloride with NaOH, followed by extraction with dichloromethane and concentration. ChB donors **D_1_**-**D_4_** were synthesized following literature.^[1-3]^ All chemicals and reagents were used as received without further purification.

**Film Preparation:** A cell was fabricated by bonding two glass substrates coated with polyvinyl alcohol (PVA, 5% aqueous solution, spin-coated at 4000 RPM for 1 min, and baked at 100 ºC for 10 min). To control the liquid crystal elastomers (LCEs) film thickness, 550 µm microspheres (ThermoScientific) were inserted as spacers. Liquid crystal mixtures were prepared by heating a blend of 1 mmol RM82, 0-0.3 mmol cysteamine, 0.4-1 mmol 1-amino-3-(dimethylamino)propane, and 0-0.5 mmol **D_n_** at 100 ºC until a clear liquid was formed. These mixtures were capillary introduced into the cells at 100 ºC and then cooled to 60 ºC. Polymerization was initiated via an Aza-Michael addition reaction, proceeding at 60 ºC for 4 hours, followed by an additional 20 hours at 90 ºC. After polymerization, the cells were opened with a blade, and strips of the desired dimensions were cut from the film.

**Monodomain Sample Preparation**: To align initially disordered LCEs, the samples were heated to 80-120 °C, uniaxially stretched (typically by 100%), and cooled to room temperature while maintaining the stretch. The aligned LCEs were then stabilized at room temperature for at least 72 hours before characterization and testing.

**Actuation Measurements:** For thermal actuation, aligned samples were placed on a black anodized aluminum sheet atop a hotplate. The temperature was increased from 30 to 120 °C in 10 °C increments. Shape changes were monitored during both heating and cooling cycles to confirm reversibility. Prior to actuation measurements, all samples underwent a complete heating and cooling cycle to remove any thermal history.

**Preparation of Light-Driven Walker:** A walking LCE device was constructed using a plastic leg connecting two sides of the LCE film. For the rolling robot model in Figure 3b, the stretched and twisted **PD_1_** film was placed directly on a hot plate.

**Preparation of different knot structure:** For obtaining the different ring structures, two ends of a twisted strip were glued together using Loctite Precision glue, for the complex structure, it also involves connecting the ends but requires winding before joining.

**Differential Scanning Calorimetry (DSC):** Conducted using a NETZSCH DSC 214 Polyma instrument with a heating/cooling rate of 10 °C/min in a nitrogen atmosphere (1 bar, 20 mL/min flow) over a temperature range of -50 to 150 °C.

**Mechanical Testing:** Stress−strain curves were obtained using a custom-built tensile tester with 500-μm-thick films stretched at 0.05 mm/s. The load–unload cycle testing was performed on the Instron 5567 universal tensile tester using a 100 N load. The tensile speed was set at 0.05 mm/s.

**Optical Characterization:** Monodomain sample alignment was examined using a polarized optical microscope (Zeiss) by imaging samples at 0° and 45° angles relative to the polarizer/analyzer axes.

**Imaging and Surface Temperature Monitoring:** Photographs and supplementary videos were captured with a Canon 5D Mark III camera (100 mm lens). Surface temperature variations were monitored using a FLIR T420BX infrared camera with a 2× close-up lens.

**Fourier Transform Infrared, Raman and NMR Spectroscopy:** Measurements were performed on a Perkin Elmer Spectrum Two spectrometer from 4000 to 650 cm^−1^ using an ATR crystal. Spectra were collected with 32 scans at a resolution of 4 cm^–1^. Raman measurements were carried out using a Thermo Scientific DXR Raman Microscope equipped with a 532 nm laser at 10 mW of power and a 10x microscope objective, resulting in a 2 µm spot size. The Raman measurements were carried out using 2.0s of exposure. ^77^Se NMR spectra of the solid film sample were recorded at room temperature using Bruker Avance 400 MHz spectrometer equipped with a SB 4 mm CPMAS probe, using 4 mm ZrO_2_ rotor. The solid sample were spun at a rate of 10 kHz. The CP contact time was 4 ms, and the relaxation delay was 5s.

**Supplementary Figures**


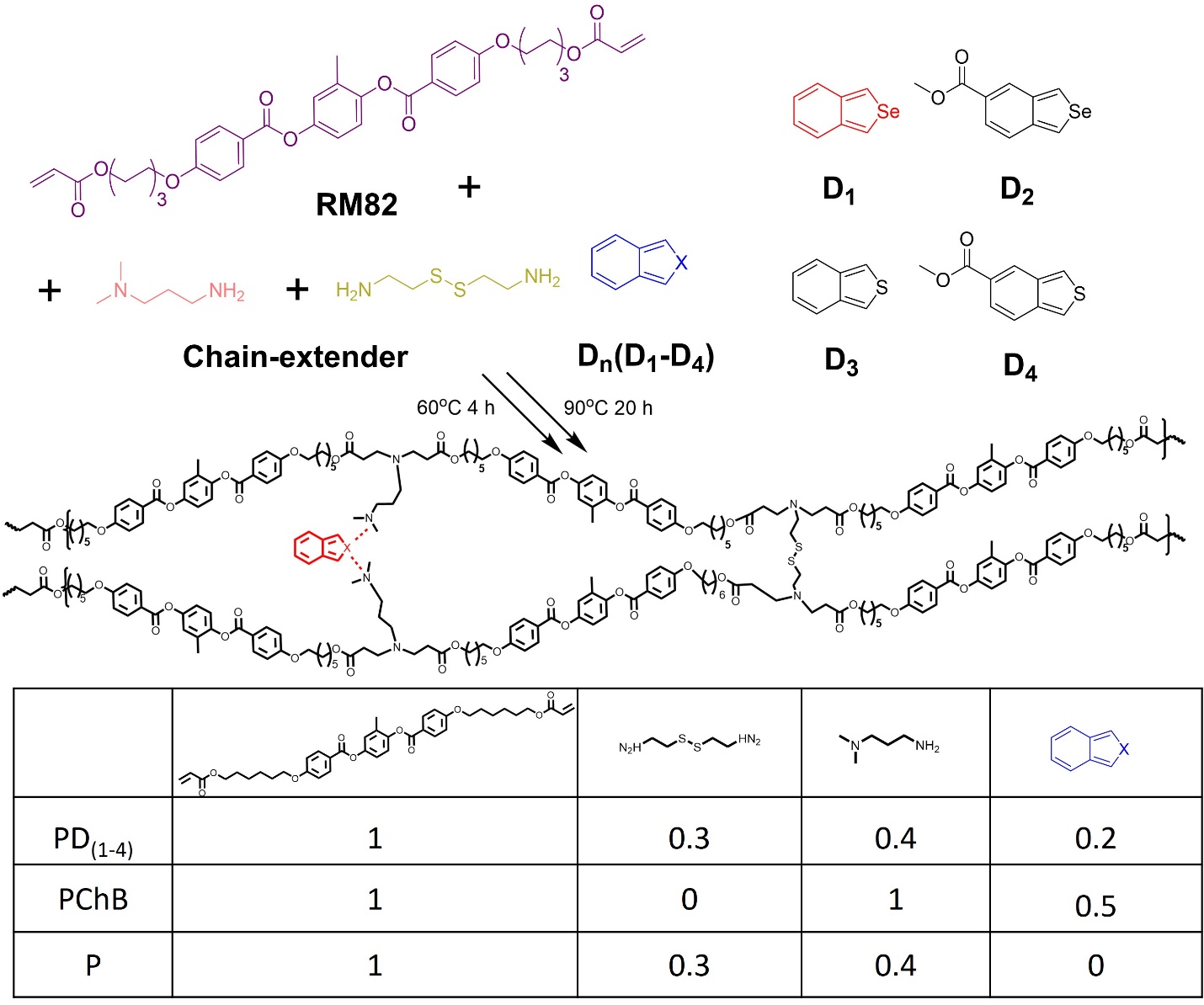


**Figure S1.** Chemical structures and synthetic routes of PDn, PChB, and the P (control) materials, along with the molar ratios of the monomers used in the preparation of PDn.


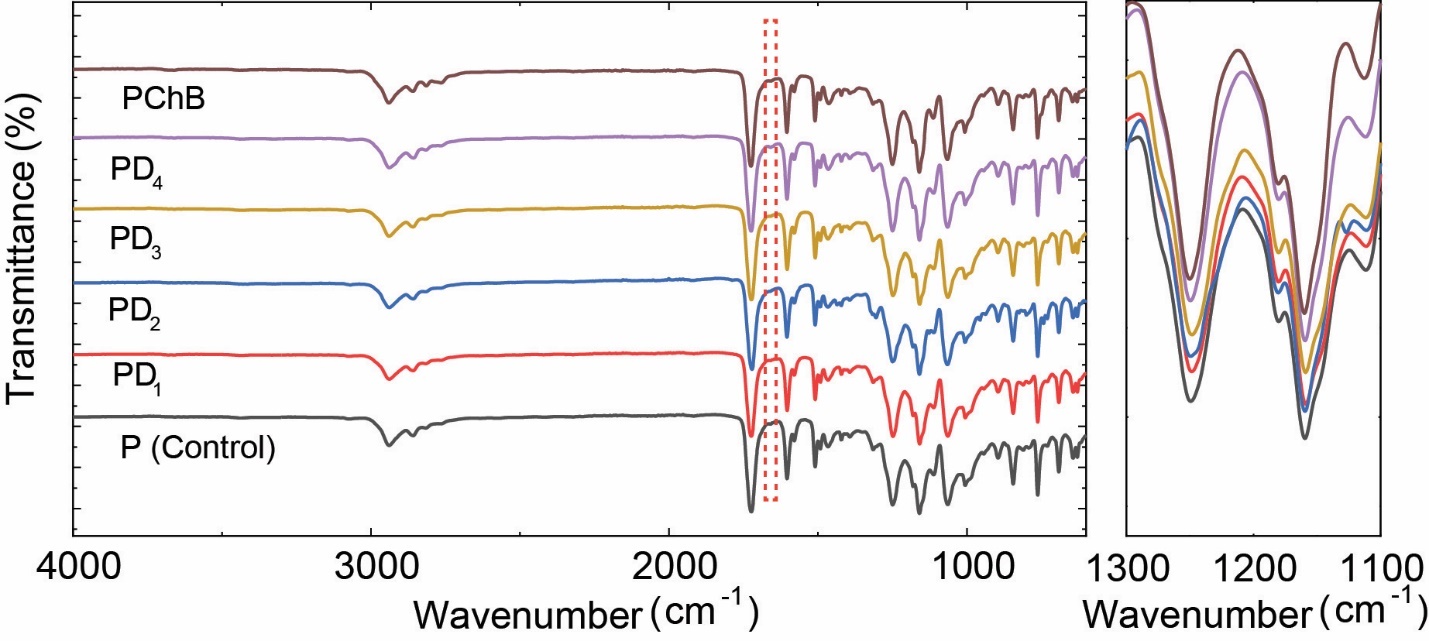


**Figure S2.** Stacked FTIR spectra of P, PChB, and PDn, highlighting the characteristic absorption bands associated with functional group transformations during ChB formation and polymer network construction.


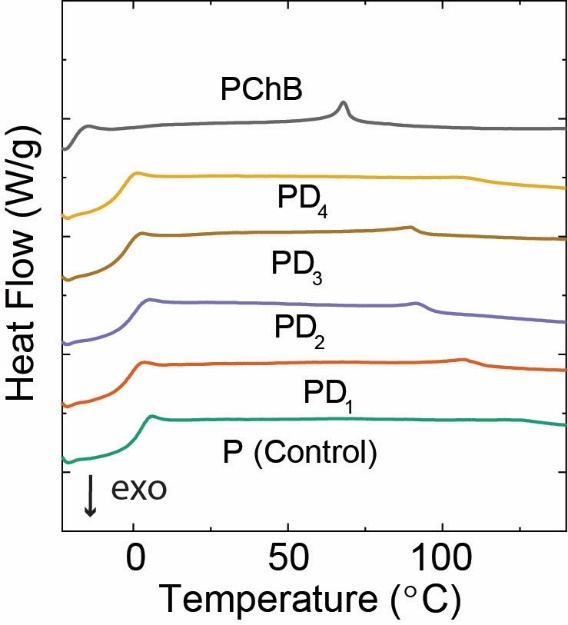


**Figure S3.** DSC curves of P, PChB and PDn. The thermal properties were analyzed using the DSC data of the second heating. Heating/cooling rate is10 ºC/min.


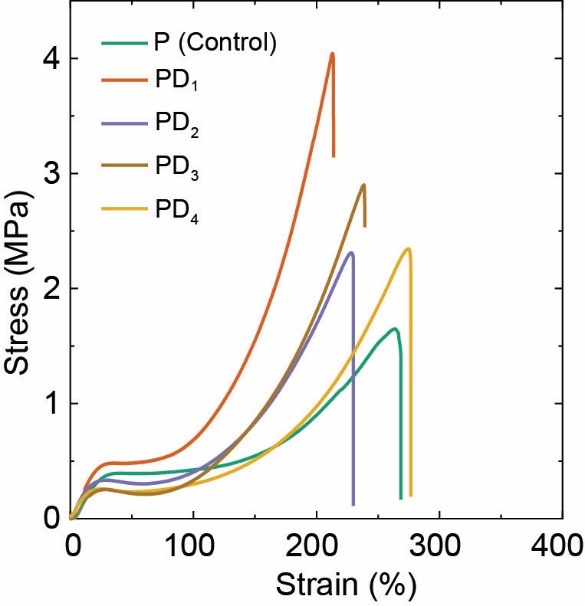


**Figure S4.** Stress–strain curves of the chalcogen-bond LCEs.


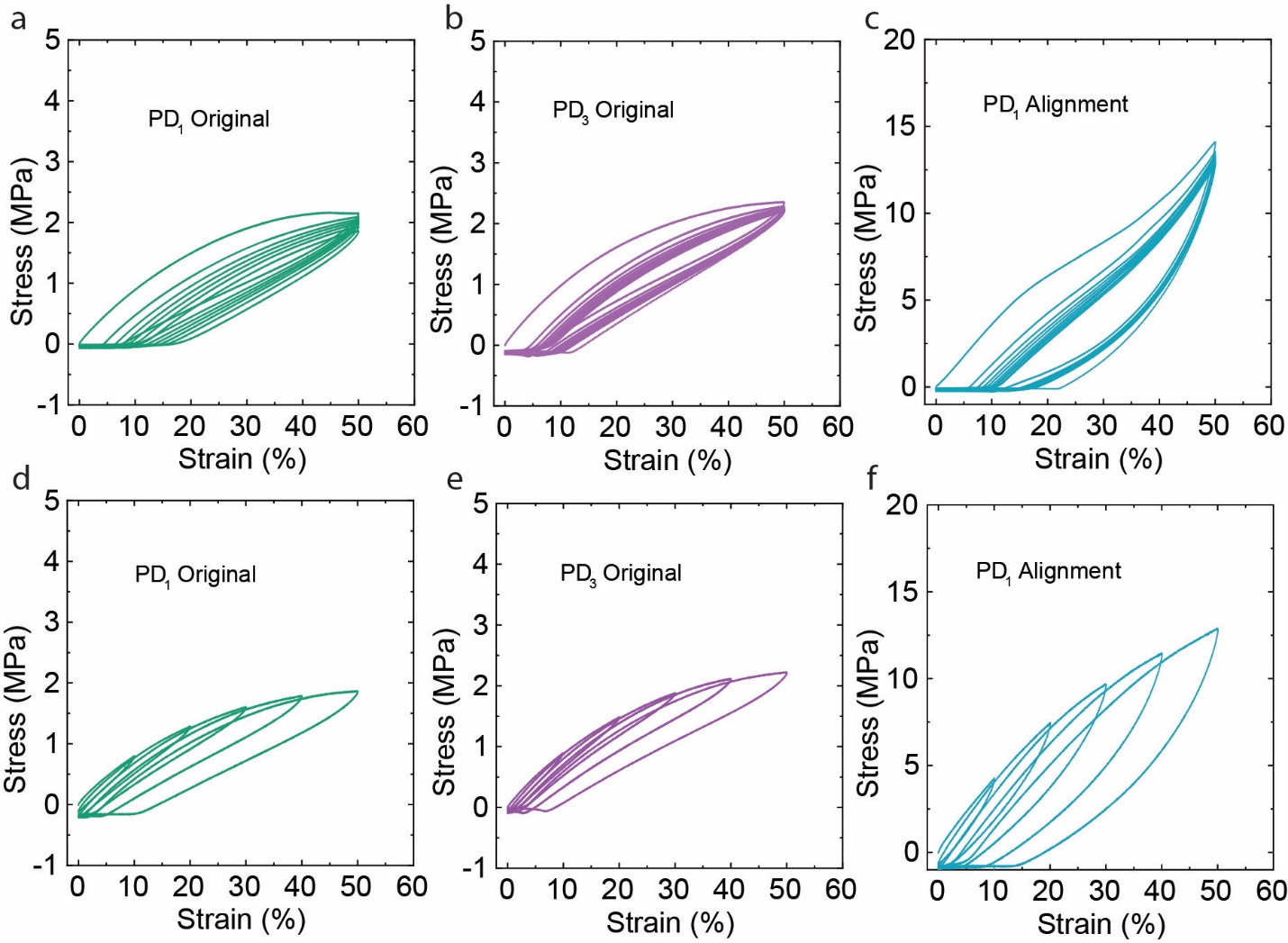


**Figure S5.** Cyclic tensile stress–strain curves of **PD_1_** and **PD_3_** before and after alignment. (a, d) **PD_1_** (original), (b, e) **PD_3_** (original), and (c, f) **PD_1_** (after alignment). The tests in (a–c) were performed in 50% strain for 7-10 cycles, while (d–f) were conducted from 10% to 50% strain.


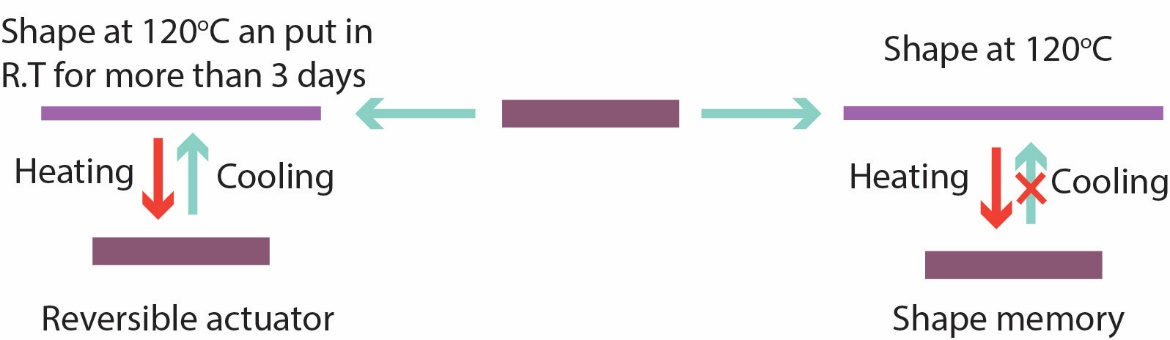


**Figure S6.** Schematic diagram of the preparation of shape memory and reversible actuators.


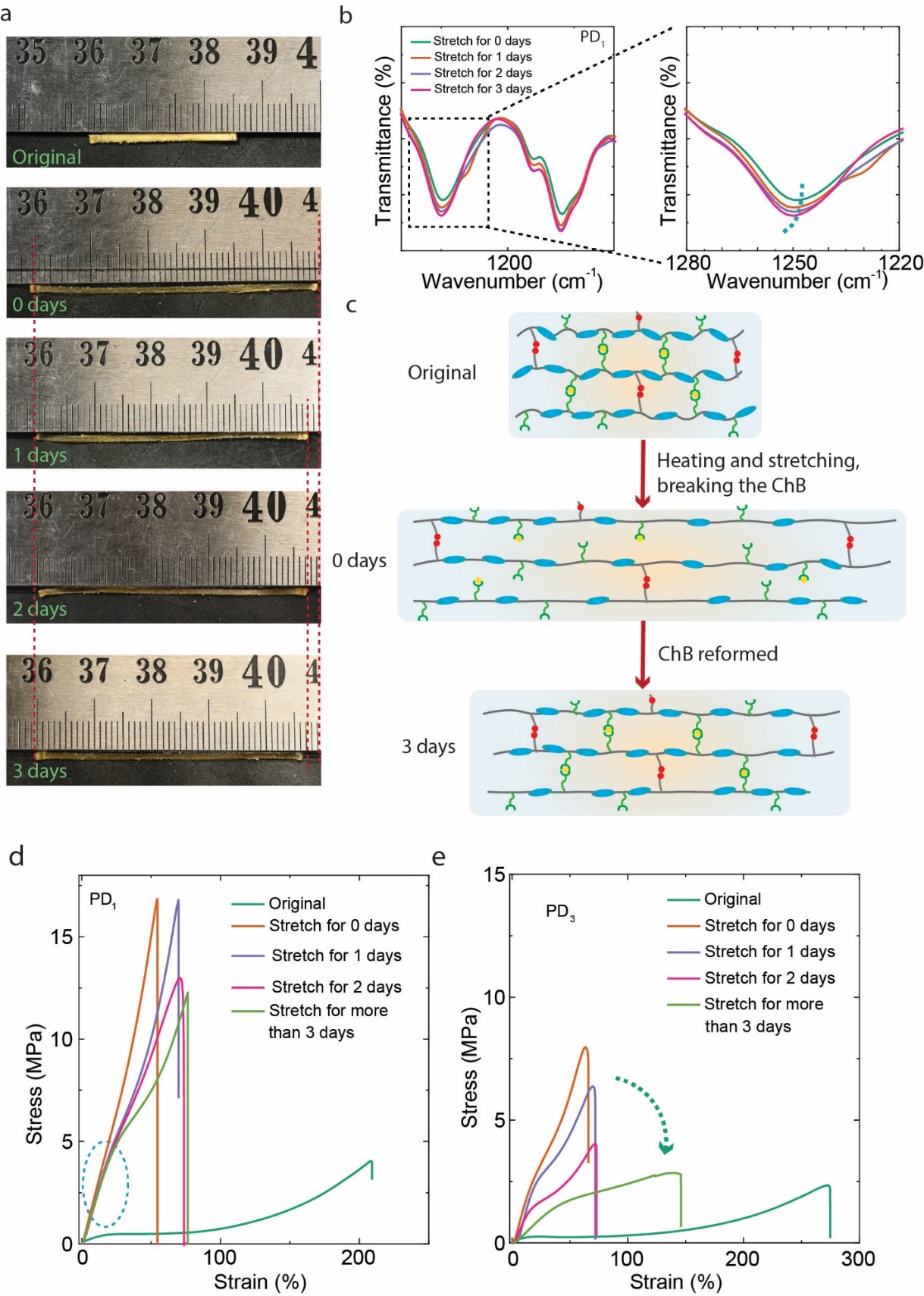


**Figure S7**. (a) Photographs of the stretching and equilibration processes of **PD_1_**. (b) FTIR spectra of the stretching and equilibration processes of **PD_1_**. (c) A schematic diagram proposing a simplified mechanism of the stretching and equilibration process. Tensile stress–strain curves of the stretching and equilibration processes of (d) **PD_1_** and (e) **PD_3._**


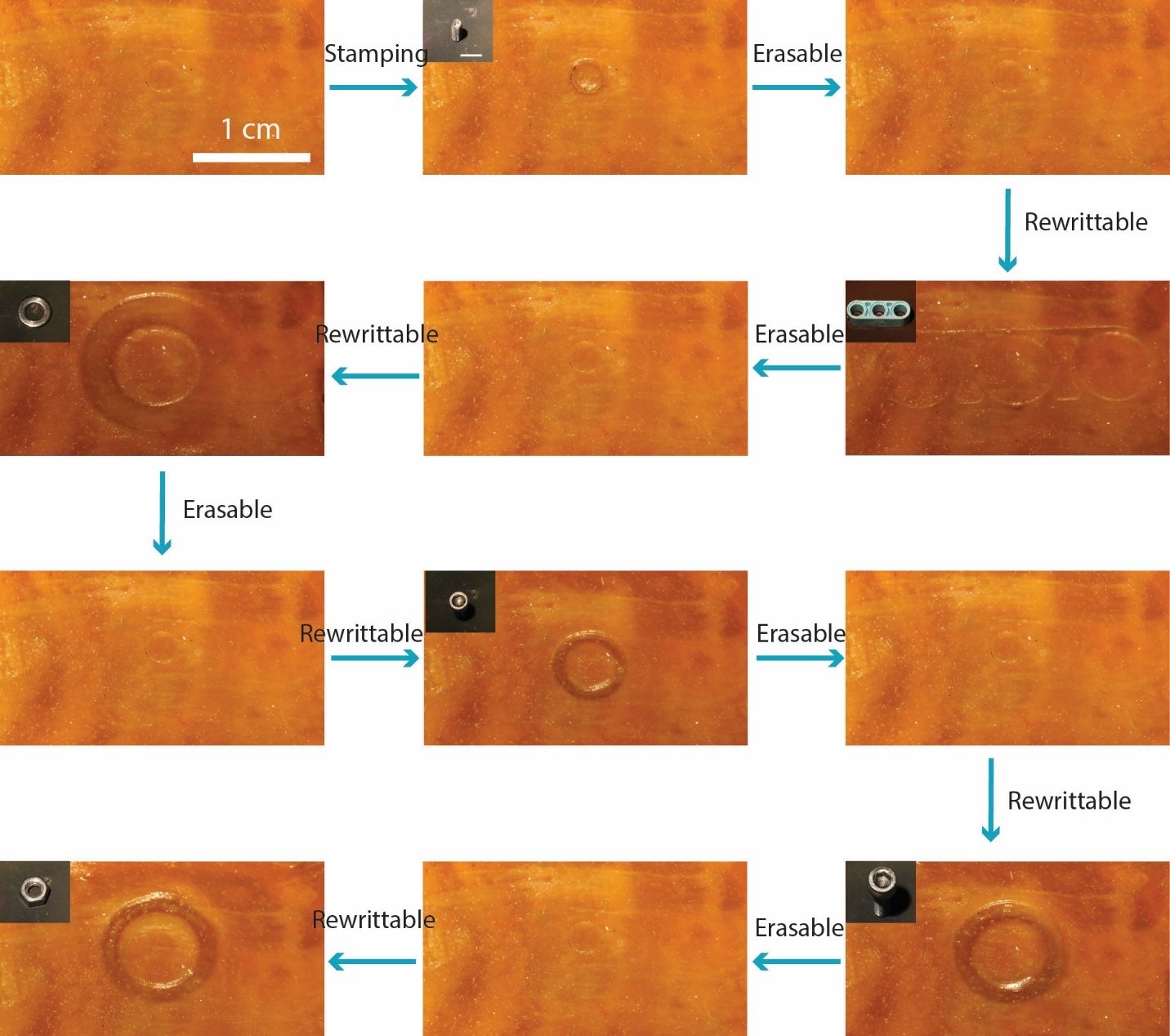


**Figure S8.** Erasable and rewritable process of shape memory properties. All scale bars are 1 cm.


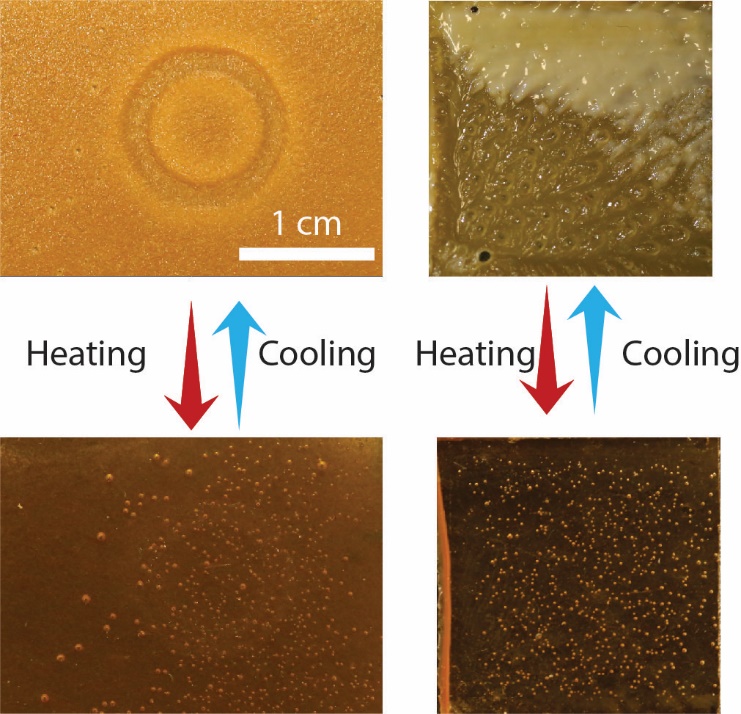


**Figure S9.** Various surface patterns demonstrating the reversible two-way shape memory effect.


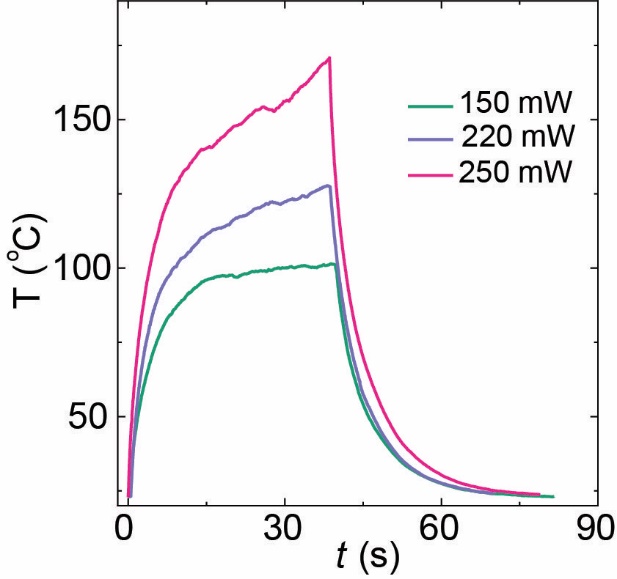


**Figure S10**. Temperature kinetic curve of PD_1_ film upon different intensity laser (532 nm) excitation.


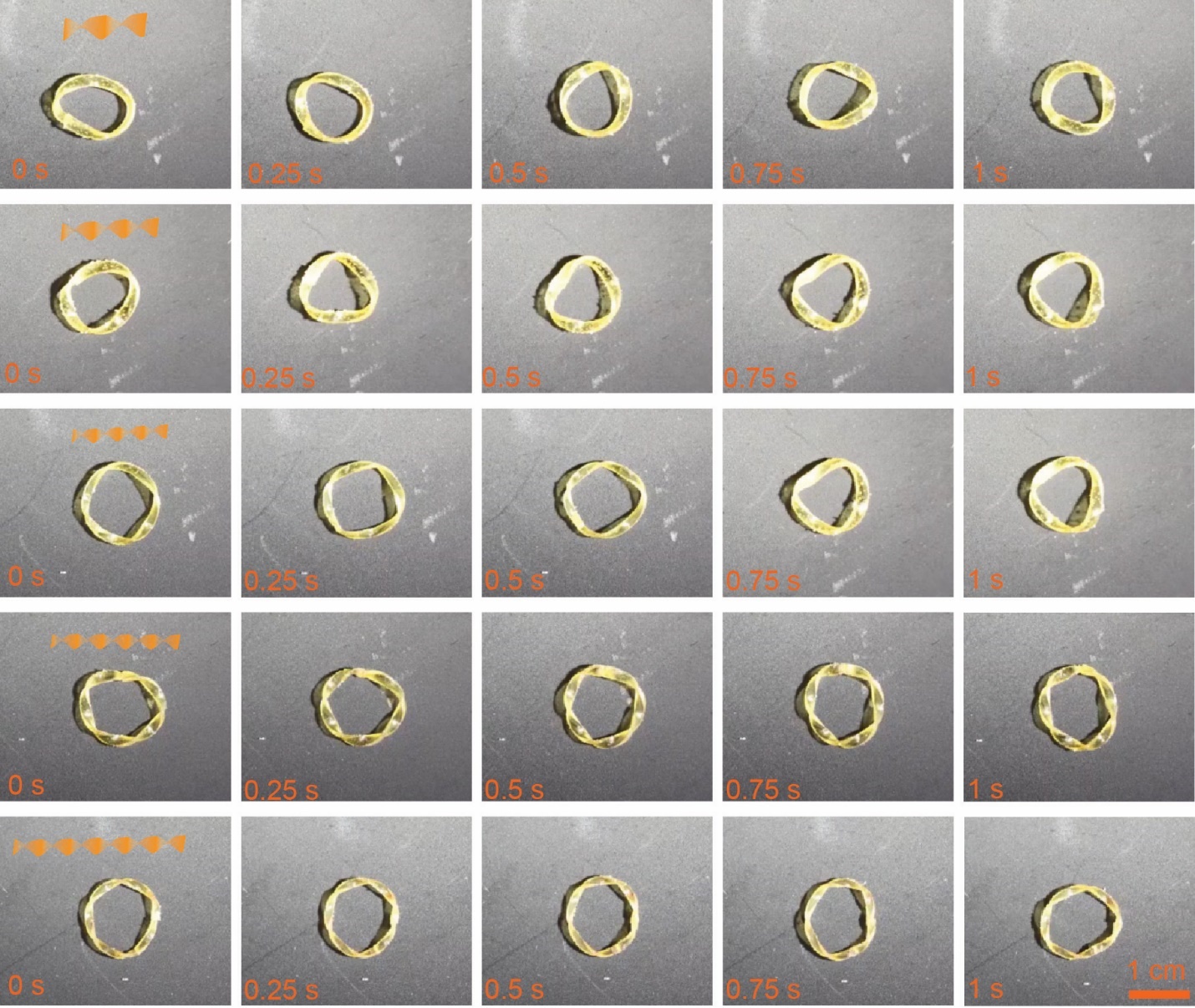


**Figure S11**. Photographs showing the self-rotating process of twist knots on a 100 °C hot plate.


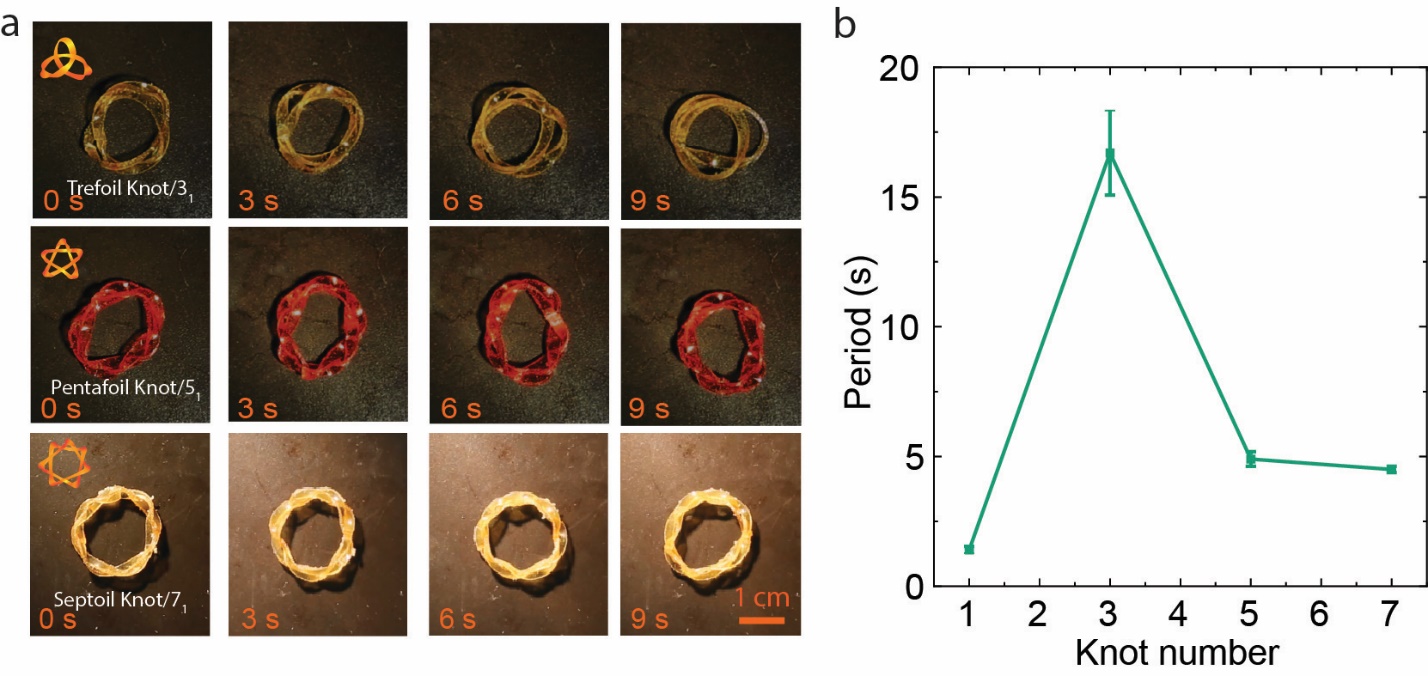


**Figure S12**. (a) Photographs showing the self-rotating process of Trefoil knot /3_1_, Pentafoil knot /5_1_ and Septoil knot /7_1_ on a 100 °C hot plate. (b) Changes in rotation period of different knot structure. The Pentafoil knot /5_1_ was dyed red.


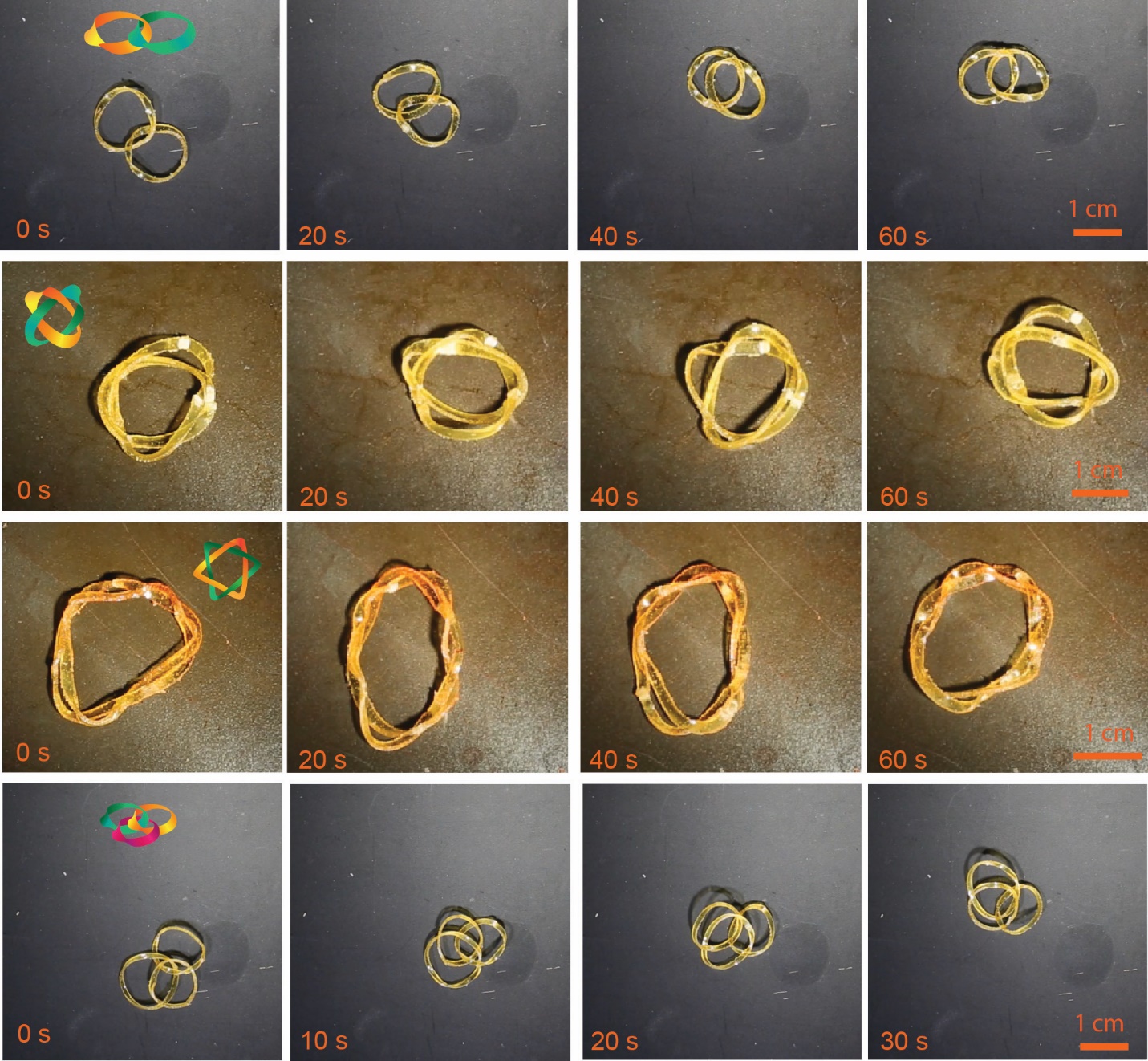


**Figure S13**. Photographs showing the self-rotating process of different links on a 100 °C hot plate.


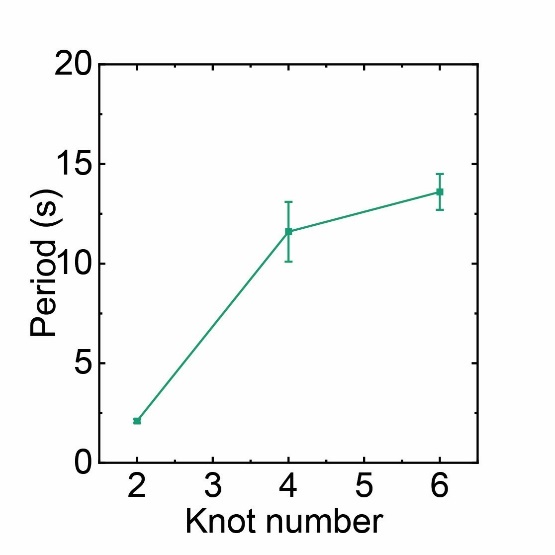


**Figure S14**. Changes in rotation period of different link structure.

**DFT details and the xyz coordinates**

**Table S1**. Computed chalcogen bond energies for **D_1_**_-_**_4_**–(NMe_2_)_2_(CH_2_)_3_ complexes at M06-2X/def2-TZVP level of theory (Ch = S, Se).

|  | Ch⋅⋅⋅N [Å] | Δ*E*_int_ [kJ mol^–1^] |
| --- | --- | --- |
| **D_1_**–(NMe_2_)_2_(CH_2_)_3_ | 2.891 | –40.2 |
| **D_2_**–(NMe_2_)_2_(CH_2_)_3_ | 2.874 | –42.4 |
| **D_3_**–(NMe_2_)_2_(CH_2_)_3_ | 2.913 | –34.3 |
| **D_4_**–(NMe_2_)_2_(CH_2_)_3_ | 2.933 | –31.7 |


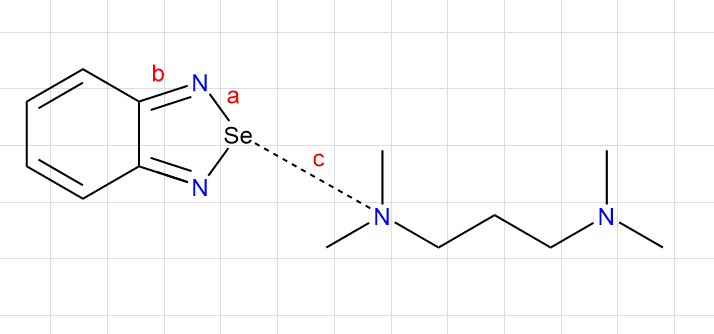


**Table S2**. DFT and experimental Raman frequencies (cm^-1^) of **D_1_**–(NMe_2_)_2_(CH_2_)_3_ complex at M06-2X/def2-TZVP level of theory.

|  | **DFT** | | **Exp** |
| --- | --- | --- | --- |
|  | Freq. (rel. intensity) | |  |
| Type | **D**_1_ | **D_1_**–(NMe_2_)_2_(CH_2_)_3_ |  |
| δ (N-C-C-C) |  | 71 (97) |  |
| c ν (Se⋅⋅⋅N) |  | 79 (90) |  |
| δ (N-C-C-C) |  | 115 (24) |  |
| δ (Me-N-C-C) |  | 129 (21) |  |
| δ (Me-N-C-C) |  | 141 (13) |  |
| δ_op_ (C-C-C-C) | 165 (9) | 174 (3) |  |
| δ (Me rotation) |  | 189 (15) |  |
| a ν_s_(Se-N_2_) | 394 (39) | 391 (70) |  |
| δ (Me-N-Me) |  | 397 (12) |  |
| δ_op_ (N-C-C-N) | 515 (2) | 517 (3) |  |
| δ (ring distortion) | 571 (24) | 574 (27) |  |
| b δ (N-Se-N) | 787 (51) | 791 (54) |  |
| δ (C-C-N) |  | 835 (14) |  |
| ν_s_(Se-N) + δ (C-C-N) | 841 (2) | 844 (3) |  |
| ν_s_(C-C) | 1009 (15) | 1011 (15) |  |
| δ_ip_ (C-C-H) | 1170 (15) | 1165 (14) |  |
| δ_ip_ (C-C-H) | 1256 (3) | 1255 (2) |  |
| ν_s_(C-N) + ν_s_(C-C) | 1356 (38) | 1350 (41) |  |
| ν_s_(C-C) | 1403 (35) | 1404 (65) |  |
| δ (H-C-H) |  | 1480 (12) |  |
| δ (H-C-H) |  | 1490 (17) |  |
| ν_s_(C-C) | 1495 (100) | 1493 (100) |  |
| δ (H-C-H) |  | 1512 (15) |  |
| ν_s_(C-C) | 1593 (40) | 1589 (30) |  |
| ν(C-H) |  | 2934 (18) |  |
| ν(C-H) |  | 2947 (28) |  |
| ν(C-H) |  | 2953 (23) |  |
| ν(C-H) |  | 2964 (35) |  |
| ν(C-H) |  | 2967 (28) |  |
| ν(C-H) |  | 2977 (69) |  |
| ν(C-H) |  | 3075 (29) |  |
| ν(C-H) |  | 3081 (11) |  |
| ν(C-H) |  | 3090 (12) |  |
| ν(C-H) |  | 3097 (14) |  |
| ν(C-H) |  | 3102 (25) |  |
| ν(C-H) |  | 3143 (11) |  |
| ν_s_(C-H) | 3220 (29) | 3217 (29) |  |
| ν_s_(C-H) | 3239 (58) | 3235 (58) |  |
|  |  |  |  |

Figure S15. Calculated Raman of **D_1_**–(NMe_2_)_2_(CH_2_)_3_ at M06-2X/def2-TZVP level of theory.

Figure S16. Calculated Raman of **D_2_**–(NMe_2_)_2_(CH_2_)_3_ at M06-2X/def2-TZVP level of theory.

Figure S17. Calculated Raman of **D_3_**–(NMe_2_)_2_(CH_2_)_3_ at M06-2X/def2-TZVP level of theory.

Figure S18. Calculated Raman of **D_4_**–(NMe_2_)_2_(CH_2_)_3_ at M06-2X/def2-TZVP level of theory.

**xyz coordinates of DFT structures**

13

D1, E= -2742.08692376 a.u.

C 0.000000000 1.432361000 -1.756163000

C 0.000000000 0.727165000 -0.509117000

C 0.000000000 -0.727165000 -0.509117000

C 0.000000000 -1.432361000 -1.756163000

C 0.000000000 -0.718787000 -2.906190000

C 0.000000000 0.718787000 -2.906190000

Se 0.000000000 0.000000000 1.876887000

H 0.000000000 2.513533000 -1.745313000

H 0.000000000 -2.513533000 -1.745313000

H 0.000000000 -1.234087000 -3.857969000

H 0.000000000 1.234087000 -3.857969000

N 0.000000000 -1.303319000 0.675004000

N 0.000000000 1.303319000 0.675004000

19

D2, E= -2969.97502930 a.u.

C 0.160230000 2.056115000 0.000007000

C 1.112207000 0.985511000 0.000003000

C 0.640384000 -0.388976000 -0.000003000

C -0.763905000 -0.656039000 -0.000006000

C -1.622921000 0.394817000 -0.000004000

C -1.157679000 1.756845000 0.000004000

Se 3.130706000 -0.480633000 -0.000001000

H 0.519756000 3.075634000 0.000014000

H -1.112305000 -1.678675000 -0.000011000

H -1.908927000 2.534939000 0.000007000

C -3.100900000 0.192271000 -0.000010000

O -3.898332000 1.091526000 -0.000013000

O -3.448981000 -1.098154000 0.000044000

C -4.853355000 -1.347630000 -0.000021000

H -5.313329000 -0.913669000 0.886461000

H -5.313332000 -0.913265000 -0.886289000

H -4.962665000 -2.427551000 -0.000191000

N 2.419448000 1.144210000 0.000006000

N 1.572116000 -1.321401000 -0.000007000

19

D3, E= -966.600479738 a.u.

C -0.531268000 1.840011000 0.000000000

C -1.602874000 0.908275000 0.000000000

C -1.334303000 -0.499763000 0.000000000

C 0.000000000 -0.973362000 0.000000000

C 1.004775000 -0.050877000 0.000000000

C 0.738667000 1.356202000 0.000000000

S -3.673601000 -0.219320000 0.000000000

H -0.742228000 2.900119000 0.000000000

H 0.219687000 -2.032062000 0.000000000

H 1.579684000 2.034448000 0.000000000

C 2.405167000 -0.569550000 0.000000000

O 2.694505000 -1.735040000 0.000000000

O 3.314496000 0.412217000 0.000000000

C 4.673626000 -0.021427000 0.000000000

H 4.877548000 -0.620240000 0.886202000

H 4.877548000 -0.620240000 -0.886202000

H 5.274035000 0.882836000 0.000000000

N -2.910165000 1.187071000 0.000000000

N -2.447464000 -1.242816000 0.000000000

13

D4, E= -738.713099144 a.u.

C 0.000000000 1.432033000 -1.237851000

C 0.000000000 0.716830000 -0.011309000

C 0.000000000 -0.716830000 -0.011309000

C 0.000000000 -1.432033000 -1.237851000

C 0.000000000 -0.714436000 -2.393195000

C 0.000000000 0.714436000 -2.393195000

S 0.000000000 0.000000000 2.235664000

H 0.000000000 2.513259000 -1.229068000

H 0.000000000 -2.513259000 -1.229068000

H 0.000000000 -1.231651000 -3.343819000

H 0.000000000 1.231651000 -3.343819000

N 0.000000000 -1.237293000 1.220243000

N 0.000000000 1.237293000 1.220243000

40

D1-Dma, E= -3129.11042016 a.u.

N 1.647349000 1.994152000 -0.203582000

N 2.257640000 -1.445647000 0.231481000

C 3.214781000 0.664373000 1.212589000

C 1.922994000 1.466495000 1.124170000

C 3.335953000 -0.461784000 0.195714000

C 2.683033000 2.893078000 -0.671759000

C 0.359202000 2.661970000 -0.219402000

C 2.054522000 -2.019035000 1.549879000

C 2.525596000 -2.488963000 -0.741654000

H 3.300439000 0.269399000 2.227096000

H 4.079335000 1.319126000 1.081512000

H 1.966265000 2.286297000 1.864759000

H 1.081922000 0.825131000 1.398745000

H 4.306310000 -0.965783000 0.350783000

H 3.346586000 -0.036176000 -0.811373000

H 3.628624000 2.366141000 -0.797589000

H 2.846437000 3.737223000 0.020703000

H 2.396566000 3.300323000 -1.641666000

H 0.356295000 3.568547000 0.408956000

H 0.099905000 2.948013000 -1.239560000

H -0.414381000 1.990914000 0.157147000

H 1.286480000 -2.789456000 1.488371000

H 2.977814000 -2.469573000 1.950059000

H 1.695852000 -1.261822000 2.244515000

H 3.452487000 -3.038846000 -0.513966000

H 1.698769000 -3.200344000 -0.762455000

H 2.625358000 -2.049213000 -1.735872000

C -3.983452000 0.351025000 -0.744071000

C -2.614404000 -0.056956000 -0.660191000

C -2.035372000 -0.352379000 0.636624000

C -2.843551000 -0.233536000 1.811485000

C -4.135128000 0.155338000 1.679627000

C -4.708019000 0.448491000 0.396793000

Se -0.218813000 -0.692806000 -1.055870000

H -4.404448000 0.568491000 -1.716275000

H -2.404227000 -0.457068000 2.774231000

H -4.759939000 0.250391000 2.558419000

H -5.745439000 0.753837000 0.351659000

N -0.765881000 -0.713874000 0.635235000

N -1.801148000 -0.195894000 -1.688481000

46

D2-Dma, E= -3356.99926389 a.u.

N -2.528485000 0.127945000 2.018302000

N -3.030834000 0.787828000 -1.404209000

C -3.519845000 2.024649000 0.732499000

C -2.325887000 1.472696000 1.500507000

C -4.025408000 1.131109000 -0.391216000

C -3.642645000 0.048918000 2.942143000

C -1.315256000 -0.351221000 2.654466000

C -2.377869000 1.951373000 -1.977948000

C -3.658335000 -0.007712000 -2.444324000

H -3.246631000 3.006757000 0.340950000

H -4.357585000 2.204670000 1.410042000

H -2.082819000 2.170890000 2.322344000

H -1.457028000 1.437465000 0.838502000

H -4.887071000 1.629672000 -0.868828000

H -4.387325000 0.190391000 0.032562000

H -4.582857000 0.272429000 2.438660000

H -3.529750000 0.745784000 3.790625000

H -3.710128000 -0.963511000 3.340814000

H -1.068300000 0.225900000 3.561184000

H -1.428638000 -1.399806000 2.932509000

H -0.473658000 -0.267069000 1.964936000

H -1.704448000 1.626670000 -2.770526000

H -3.104382000 2.664922000 -2.400001000

H -1.772595000 2.458213000 -1.228746000

H -4.450969000 0.548455000 -2.968952000

H -2.912281000 -0.318231000 -3.177292000

H -4.103233000 -0.903227000 -2.006139000

C 2.441470000 -2.410240000 0.259059000

C 1.189504000 -1.829993000 -0.121665000

C 1.124396000 -0.414848000 -0.426733000

C 2.307020000 0.380488000 -0.347758000

C 3.471859000 -0.218243000 0.017537000

C 3.538546000 -1.620985000 0.322176000

Se -1.197257000 -1.309927000 -0.699224000

H 2.480296000 -3.466951000 0.485184000

H 2.257940000 1.435203000 -0.577439000

H 4.502811000 -2.021424000 0.604166000

C 4.742868000 0.552156000 0.121275000

O 5.796696000 0.069743000 0.441312000

O 4.594491000 1.848154000 -0.179893000

C 5.784132000 2.628617000 -0.095852000

H 6.184343000 2.600228000 0.916563000

H 6.536717000 2.246122000 -0.783768000

H 5.495812000 3.639748000 -0.366205000

N 0.048979000 -2.482763000 -0.226208000

N -0.065127000 0.054195000 -0.761297000

46

D3-Dma, E= -1353.62188613 a.u.

N -2.611761000 1.767719000 -0.689558000

N -3.309964000 -1.543871000 0.137207000

C -3.932591000 -0.053663000 -1.778205000

C -2.638058000 0.747428000 -1.727749000

C -4.331939000 -0.691637000 -0.454678000

C -3.674002000 2.742730000 -0.838134000

C -1.321957000 2.432469000 -0.675804000

C -2.866465000 -2.603891000 -0.747264000

C -3.787157000 -2.089641000 1.392186000

H -3.825892000 -0.818530000 -2.550370000

H -4.760336000 0.578618000 -2.107225000

H -2.466418000 1.202955000 -2.720137000

H -1.803749000 0.068565000 -1.532990000

H -5.264836000 -1.263545000 -0.608507000

H -4.550079000 0.096455000 0.271190000

H -4.650630000 2.277233000 -0.709108000

H -3.651788000 3.234411000 -1.826103000

H -3.567013000 3.512225000 -0.073281000

H -1.151104000 3.022850000 -1.591518000

H -1.253982000 3.099137000 0.184868000

H -0.521656000 1.693883000 -0.603395000

H -2.144437000 -3.227892000 -0.221443000

H -3.701852000 -3.239140000 -1.086836000

H -2.362236000 -2.190719000 -1.619575000

H -4.664384000 -2.743257000 1.256915000

H -2.996488000 -2.671845000 1.868543000

H -4.068228000 -1.277414000 2.065048000

C 2.320824000 1.224859000 1.865561000

C 1.071387000 0.609447000 1.596666000

C 0.958216000 -0.367535000 0.558773000

C 2.090502000 -0.730118000 -0.206194000

C 3.279765000 -0.120525000 0.078336000

C 3.394637000 0.857287000 1.114853000

S -1.187864000 -0.099916000 1.514163000

H 2.397364000 1.962613000 2.652128000

H 2.023095000 -1.467447000 -0.994391000

H 4.362433000 1.303551000 1.292452000

C 4.461167000 -0.517608000 -0.740827000

O 4.432116000 -1.329651000 -1.626047000

O 5.576162000 0.133500000 -0.383954000

C 6.740631000 -0.205814000 -1.133169000

H 6.963453000 -1.266152000 -1.024552000

H 6.588541000 0.016577000 -2.188251000

H 7.544015000 0.399462000 -0.724793000

N -0.288327000 -0.845908000 0.422289000

N -0.093368000 0.830992000 2.218434000

40

D4-Dma, E= -1125.73362070 a.u.

N 1.466907000 1.914539000 -0.056854000

N 2.302669000 -1.467321000 -0.072768000

C 3.211251000 0.550379000 1.100007000

C 1.862414000 1.253688000 1.178067000

C 3.336441000 -0.442137000 -0.047735000

C 2.413162000 2.929495000 -0.474741000

C 0.144589000 2.494024000 0.087754000

C 2.223784000 -2.229873000 1.157344000

C 2.502418000 -2.344465000 -1.208758000

H 3.387298000 0.050254000 2.054788000

H 4.017951000 1.280146000 1.000575000

H 1.890150000 1.978874000 2.012155000

H 1.088499000 0.517703000 1.410908000

H 4.337318000 -0.908581000 -0.001461000

H 3.273008000 0.098474000 -0.995915000

H 3.376819000 2.485359000 -0.722532000

H 2.573932000 3.694707000 0.304656000

H 2.035975000 3.428523000 -1.367661000

H 0.135399000 3.327283000 0.810568000

H -0.207965000 2.866261000 -0.875031000

H -0.558368000 1.738680000 0.442896000

H 1.468910000 -3.007824000 1.046431000

H 3.185592000 -2.704725000 1.415639000

H 1.913015000 -1.593181000 1.984473000

H 3.455032000 -2.896218000 -1.148479000

H 1.689092000 -3.069883000 -1.265319000

H 2.504829000 -1.759782000 -2.130407000

C -2.904990000 -0.749486000 1.536260000

C -2.056429000 -0.614910000 0.407011000

C -2.548665000 -0.017311000 -0.796063000

C -3.887078000 0.447332000 -0.869152000

C -4.674922000 0.302241000 0.232195000

C -4.184618000 -0.294790000 1.432278000

S -0.280522000 -0.600087000 -1.157356000

H -2.525986000 -1.202679000 2.441980000

H -4.249499000 0.897506000 -1.783059000

H -5.700998000 0.645382000 0.206520000

H -4.855113000 -0.384100000 2.277145000

N -1.621008000 0.030530000 -1.760379000

N -0.772952000 -0.990021000 0.317436000

**References.**

[1] B. A. DaSilveira Neto, et al., Photophysical and electrochemical properties of π-extended molecular 2,1,3-benzothiadiazoles. *Tetrahedron* **2005**, *61*, 10975.

[2] B. Gadakh, et al., Base substituted 5′-O-(N-isoleucyl)sulfamoyl nucleoside analogues as potential antibacterial agents. *Biorg. Med. Chem.* **2014**, *22*, 2875.

[3] J. Alfuth, et al., Hydrogen and chalcogen bonds in crystals of chalcogenadiazolecarboxylic acids – competition or cooperation? *CrystEngComm* **2024**, *26*, 2918.

**Captions for Videos**

**Video S1.** **PD_1_** film actuator as a self-sustained roller on a 100 °C hot plate. Actuator dimensions: 24 × 1 × 0.4 mm^3^.

**Video S2.** **PD_1_** film actuator as a walker upon modulated laser light excitation. Light: 532 nm, 800 mW. Actuator dimensions: 20 × 2 × 0.4 mm^3^.

**Video S3.** Self-oscillation of a single ring with different twist numbers. This video shows continuous oscillations of a single ring with different twist numbers. The temperature of the hotplate is 100°C. The movie is played with 4× accelerated speed.

**Video S4.** Self-oscillation of knot structure ring. This video shows continuous oscillations of a complex knot structure ring. The temperature of the hotplate is 100°C. The movie is played with 4× accelerated speed.

**Video S5.** Self-oscillation of two link rings. This video shows continuous oscillations of two link rings. The temperature of the hotplate is 120°C. The movie is played with 4× accelerated speed.
